# Boring life: early colony formation and growth in the endolithic bryozoan genus *Penetrantia* Silén, 1946

**DOI:** 10.1186/s40851-024-00234-z

**Published:** 2024-06-14

**Authors:** Sebastian H. Decker, Sarah Lemer, Simone Decker, Masato Hirose, Mildred J. Johnson, Thomas Schwaha

**Affiliations:** 1 Department of Evolutionary Biology, University of Vienna, Schlachthausgasse 43, Vienna, 1030 Austria; 2 Marine Laboratory, UOG Station, Mangilao Guam, 96923 USA; 3 School of Marine Biosciences, Kitasato University, Kitasato, Minami-ku, Sagamihara City, Kanagawa 252-0373 Japan

**Keywords:** Boring bryozoans, Stolonate ctenostomes, Growth rates, Brooding, Bioerosion

## Abstract

As in most colonial and sessile marine invertebrates, bryozoan life history is characterized by asexual propagation of zooids for colonial growth and by sexual production of larvae for dispersal. However, comprehensive life histories, particularly in cryptic species such as endolithic (boring) bryozoans, remain poorly understood. The ctenostome family Penetrantiidae is widespread from temperate to tropical waters and often found in molluscan shells, offering an opportunity to study the boring lifestyle and its potential impact on bioerosion through growth and settlement experiments. Our research focused on *Penetrantia clionoides* from Guam in the Pacific Ocean, *Penetrantia japonica* from Japan, and a *Penetrantia* species from France in the Atlantic Ocean. We found distinct life histories and reproductive patterns potentially influenced by environmental factors such as temperature and food availability. The tropical *P. clionoides* displayed higher rates of larval production and growth compared to its temperate counterpart. For instance, the mean stolon extension was 335.2 μm/week in *P. clionoides* versus 232.1 μm/week in *Penetrantia* sp. Autozooid development took 13 days in *P. clionoides* and 31 days in *Penetrantia* sp. Anatomical features like apertural rims aided in species identification and in understanding larval settlement preferences, suggesting a tendency for philopatric settlement behavior. The bioerosional impact of penetrantiids remains little understood, but we generated first projections of bioerosion rates and a protocol for keeping *Penetrantia* under laboratory conditions, laying a foundation for further research in this field.

**Supplementary Information:**

The online version contains supplementary material available at 10.1186/s40851-024-00234-z.

## Background

The life history of colonial and sessile animals is often complex and characterized by asexual, clonal reproduction for colony growth and habitat colonization, and sexual production of mobile larvae for dispersal [1–4]. That also applies to the colonial lophotrochozoan phylum Bryozoa, which comprises about 6,000 extant species [3, 5]. The majority of all bryozoans live in marine environments, with more than 5,500 species belonging to the clade Gymnolaemata [5]. This group is further subdivided into the calcifying Cheilostomata and the uncalcified and paraphyletic “Ctenostomata” [6, 7], with the latter group displaying a high morphological plasticity including many cryptic members with highly specialized lifestyles, e.g., endolithic forms [8]. Endolithic bryozoans are often referred to as boring bryozoans, since they are capable to penetrate into hard substrates made of biogenic carbonate. All endolithic bryozoans apply a chemical mechanism to dissolve calcium carbonate, lacking specialized gnawing organs for a mechanical excavation method [9, 10]. Four Recent families of ctenostome bryozoans (Terebriporidae, Spathiporidae, Immergentiidae, and Penetrantiidae) have evolved such a boring lifestyle [8, 9]. The family Penetrantiidae is monogeneric, including 11 extant species, which occur in temperate or tropical waters and most commonly found in the shells of mollusks [9, 11].

Similarly, to other bryozoans, boring bryozoan colonies are a mosaic of genetically identical units called zooids. Individual zooids are compartmentalized into two main structural parts, the cystid and the polypide [7, 12]. The cystid largely represents the body wall while the polypide is placed inside the protective cystid and includes major organs like the digestive system and the lophophore [7, 12]. The lophophore, shaped like a crown of tentacles, is the filter-feeding apparatus in bryozoans and the only part protruding into the water column in boring bryozoans, while the rest of the zooid is completely immersed within calcareous substrates [7, 8, 12–14]. Bryozoan colony growth results from asexual budding of new zooids, creating encrusting or erect colonies on predominantly hard substrates [3]. Many bryozoans are known to produce non-feeding polymorphic zooids with different functions in a colony and are referred to as heterozooids, while feeding zooids are called autozooids [12, 15]. In Penetrantiidae, the stolonal network is composed of polymorphic kenozooids, which play a crucial role in colony growth by extending the colony horizontally to the surface of the substrate and producing autozooids or additional stolons at regular intervals, effectively interconnecting the entire colony [8, 11]. The stolons in endolithic bryozoans feature unique tubulets, which are small tubes that extend towards the substrate surface and create small pores (about 2 μm) within it. They might act as spacers to ensure the colony is placed at a constant depth within the substrate, but their exact function remains unclear [9, 11]. In Penetrantiids, each zooid is connected by a short peduncle to its stolonal network, which is part of the zooid until a septum marks the transition into the corresponding stolon [11, 13]. Penetrantiid gonozooids are specialized polymorphs for reproduction with unique brood chambers for embryo incubation [11, 14]. Since the gonozooids in most species lack a functional polypide including a digestive tract, they are probably not capable of feeding and thereby considered true heterozooids [9, 11, 14]. Penetrantiids are considered colonial hermaphrodites, in which some autozooids function as males and gonozooids as females [11, 16]. After fertilization, a single zygote is transferred from the zooidal tube of a gonozooid into its brood chamber and develops into a short-lived lecithotrophic larva of the coronate type [11]. During their short pelagic stage, bryozoan larvae scan substrates for a suitable place to settle and metamorphose into an ancestrula, which is the founder zooid of each colony and marks the onset of asexual zooid propagation for colony growth [3, 17].

Since boring bryozoans have to dissolve their substrate during settlement and colony growth, they contribute to bioerosion and are considered internal microborers [18, 19]. The role of boring bryozoans in bioerosion processes remains a mystery, much like the lack of information regarding their general life history, including growth rates, reproduction, and larval behavior [19].

To date, the complete life history of only a few bryozoan species has been investigated in detail and they showed highly diverse reproductive patterns, colony growth forms and larval preferences, often adapted to specific substrate types [3, 17, 20–24].

Observations of live penetrantiids are essential for filling the knowledge gap regarding this peculiar lifestyle in bryozoans. We conducted one of the first growth and settlement experiments on endolithic bryozoans, comparing two different penetrantiid species from distinct geographic and bathymetric regimes. To test whether different environmental parameters affect reproduction and colony growth rates, we selected the tropical *Penetrantia clionoides* from Guam in the Pacific Ocean and the temperate *Penetrantia* sp. from Roscoff, France, in the North Atlantic Ocean as study organisms. Additionally, we gained new insights to colony formation and larval recruitment patterns of the recently described *Penetrantia japonica* from Japan.

## Methods

### Sample collection

Three different penetrantiid species were investigated for this study. Live specimens of *Penetrantia clionoides* were collected by hand in the intertidal zone of Pago Bay, Guam (13°25.655’N 144°47.890’E) in November and December 2022. Live specimens of *Penetrantia* sp. were collected by dredging around Stolvezen close to Roscoff, France (48°42.847’N 3°53.5’W) in August to September 2021 and additional specimens in December 2022 and March 2023 for further investigations in Vienna. Specimens of *Penetrantia japonica* were only used for histological investigation and were collected by hand in the intertidal zone of Sagami Bay, Japan (35°13.336’N 139°36.152’E) in August 2023. Specimens were fixed either in 96% ethanol or 2% glutaraldehyde and stored at 4 °C until further investigations.

### Experimental setup

Colony growth and settlement experiments were carried out only with *P. clionoides* from Guam and *Penetrantia* sp. from Roscoff, France. Experiments on live *P. clionoides* were conducted at the Marine Laboratory of the University of Guam in Mangilao, Guam.

Experiments on live *Penetrantia* sp. were conducted at the Station Biologique de Roscoff in Roscoff, France. To document growth rates of mature colonies, shells with live colonies were selected and kept in large seawater tanks (225 L in Guam, 25 L in Roscoff) with constant exchange of unfiltered seawater for four weeks. Colonies of *P. clionoides* were in alive shells of the gastropod *Drupa morum*. The colony of *Penetrantia* sp. was in a dead shell of the bivalve *Anomia ephippium*. Translucent shell parts were reduced in size into smaller fragments to facilitate better documentation. In total, two colonies of *P. clionoides* and one colony of *Penetrantia* sp. were considered suitable for documentation and the corresponding shell fragments were placed in Petri dishes (diameter 3 cm) and fixed with the Coralscaper gel MICROBE-LIFT (Ecological Laboratories, Inc., Cape Coral, Florida, USA) to ensure daily photo documentation was performed consistently (Fig. S1). Colonies of *Penetrantia* sp. from France were fed daily with a 10 ml mixture of the microalgae *Tisochrysis lutea* and the diatom *Chaetoceros calcitrans*, since the station in Roscoff had a suitable culture of microalgae available. Water temperature was monitored constantly and fluctuated between 28 and 30 °C in the Guam setup (November and December 2022) and between 14 and 18 °C in the Roscoff setup (September - October 2021).

For settlement experiments, 10 pristine and translucent shell fragments (*A. ephippium* or *D. morum*) with no boring traces were placed in small containers within the sea water tanks. Ten shell fragments with mature colonies were selected and placed among the pristine shell fragments for four weeks. Every second day, the pristine shell fragments were checked for any signs of settled larvae. If a shell fragment was colonized by a larva it was glued into a Petri dish (diameter 3 cm) and its growth documented daily.

### Documentation and imaging

Stereomicroscopic images of live colonies were obtained with a Nikon SMZ stereomicroscope (Nikon, Tokyo, Japan) combined with a Nikon Z6 mirrorless camera. Images of fixed specimens were taken either with a Nikon SMZ25 stereomicroscope using a DsRi2 microscope camera, or with a Hirox RH−2000 3D digital microscope (Hirox Co., Ltd., Tokyo, Japan).

Scanning electron microscopic imaging and element analysis were carried out with dry samples of *P. clionoides* and *P. japonica* using a JEOL IT 300 (JEOL, Akishima, Tokyo, Japan) scanning electron microscope with either a secondary or backscattered electron detector at 10–25 KeV. For imaging, samples were gold sputtered for 120 s with a JEOL JFC−2300 h sputter coater while samples for element analysis were left uncoated.

The shell piece containing *Colony 1* of *P. clionoides* which was observed during the growth experiments for four weeks, was fixed in 2% glutaraldehyde and used for computer tomographic (CT) scans. First, it was bleached and then dried in an ethanol series. A Bruker SkyScan 1272 (Bruker, Billerica, Massachusetts, USA) was used to obtain micro-CT scans. An overview scan was obtained at a voltage of 100 kV and reconstructed with 11.9 μm voxel size, followed by detailed scans at a voltage of 100 kV and 3.9 μm voxel size. The reconstructed tomographic images were further processed in Amira v. 2020.2 (FEI, Oregon, USA). Shell and boring traces were segmented and visualized as surface renderings. The volume eroded by *Colony 1* of *P. clionoides* within the four weeks of observation was calculated using the label analysis tool within Amira.

### Data analyses

For *P. clionoides* and *Penetrantia* sp., stolonal growth was measured weekly based on stereomicroscopic images using Photoshop (Adobe Inc., San Jose, CA, USA). The weekly mean was calculated for each individual stolon, for all stolons within one colony, and for all stolons within one species. Daily mean is based on the total length extension of the corresponding stolon within the observed time. Stolon growth of ancestrulae was not included in the overall mean of the corresponding species.

To estimate reproductive rates, 10 shell fragments with mature colonies were collected within a single month and decalcified in 20% ethylenediaminetetraacetic acid (EDTA). In extracted colonies, zooids were counted in a defined area of 0.1 cm^2^ and the ratio between autozooids, gonozooids and brooding gonozooids was calculated.

## Results

### Reproductive patterns

**Fig. 1:**
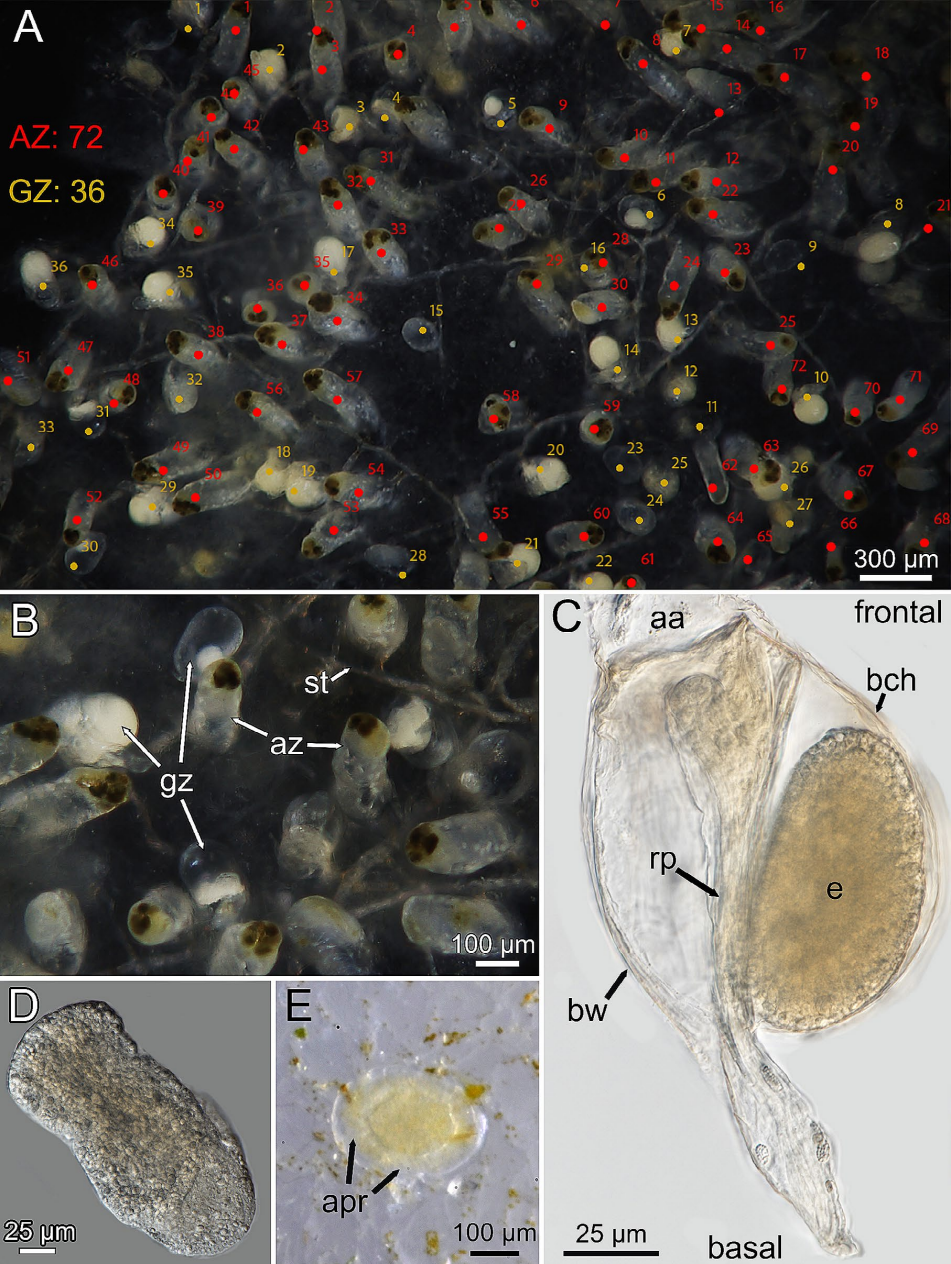
Reproduction in *Penetrantia clionoides* from Guam. **A** Overview image of a decalcified colony within the area of 0.1 cm², including count of autozooids (AZ) in red and gonozooid (GZ) in yellow. **B** Close-up of autozooids and brooding gonozooids with embryos of same colony shown in A. **C** Microscopic images of a gonozooid from a lateral perspective. **D** Extracted embryo. **E** Preancestrula two days post settling. *Abbreviations* aa – apertural area, apr – apertural rim, az – autozooid, bha – bore hole aperture, bch – brood chamber, bw – body wall, e – embryo, gz – gonozooid, rp – reduced polypide, st -stolon

In 10 colonies of *Penetrantia clionoides* collected in November and December 2022, gonozooids with brooding embryos were always present. For one colony the ratio of brooding gonozooids and autozooids was estimated: in an area of 0.1 cm^2^ the colony contained 108 zooids: 72 autozooids and 36 gonozooids, 30 of 36 gonozooids were brooding an embryo which equals 27% of all zooids in the examined area (Fig. 1A). All autozooids contained several brown bodies, indicating many polypide regeneration cycles, while not a single gonozooid contained a brown body (Fig. 1A–C).

In contrast, 10 colonies of *Penetrantia* sp. from France collected in August and September 2022, contained no brooding embryos. In March 2023, two of 10 colonies contained one brooding gonozooid. For one colony the ratio of brooding gonozooids and autozooids was estimated: in an area of 0.1 cm² the colony contained 53 zooids: 52 autozooids and one brooding gonozooid, which equals 1.9%.

### Larval settlement and preferences

**Fig. 2:**
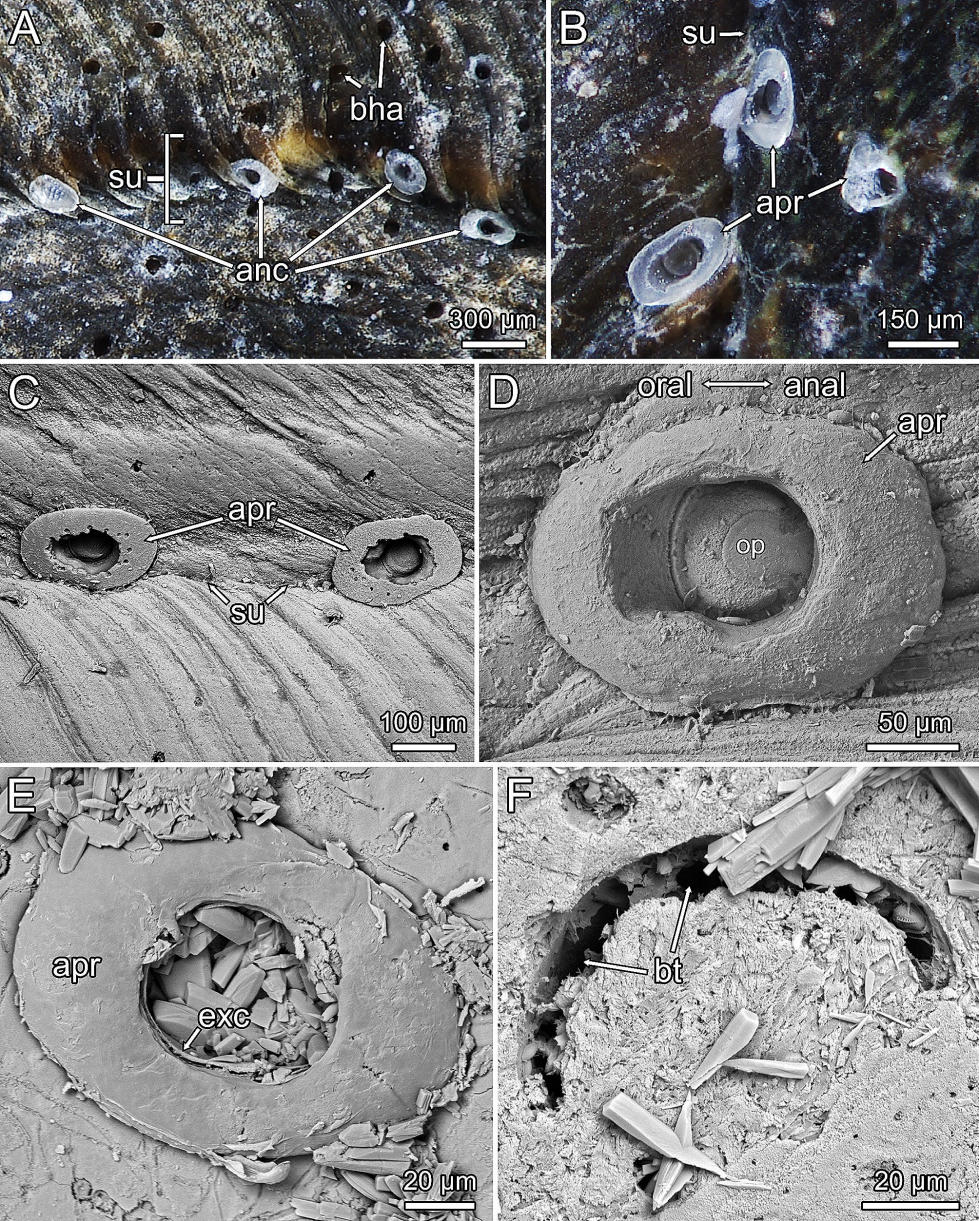
Borehole apertures with apertural rim in *Penetrantia japonica* from Japan **A–D** and *Penetrantia clionoides* from Guam **E–F**. **A–B** Stereomicroscopic images of *P. japonica* with prominent apertural rims sitting in the sutures of shells of the gastropod *Tegula rugata*. **C–D** Scanning electron microscopic (SEM) images of apertural rims in *P. japonica*. **E** SEM images of the apertural rim in *P. clionoides*. **F** SEM image of developing borehole aperture of an autozooid in *P. clionoides*. *Abbreviations* anc – ancestrula, apr – apertural rim, bha – bore hole aperture, bt – boring trace, exc – exterior cuticle, op – operculum, su – suture of gastropod shell

Neither free-swimming larvae nor the actual settlement of larvae were observed in this study and thereby our data corresponds to the recruitment pattern of successfully settled larvae that have already metamorphosed. Based on the appearance of two new preancestrulae after only a day from the start of the settlement experiment with *P. clionoides*, it is clear that the free-swimming phase is short and that larvae settle immediately or shortly after release (Fig. 1D, E). In *P. clionoides* and *Penetrantia japonica* larvae tend to settle in close proximity to each other and to the parent colony if more space is available (Fig. 2A–C). In *P. japonica* most ancestrulae were observed within shell sutures (gap between shell whorls), as in the gastropod *Tegula rugata* (Fig. 2A–D). In *P. clionoides*, the preancestrula is elliptical with flat edges that rise towards the center and measures approximately 250 μm in length, 185 μm in width and 15 μm in height (*n* = 6) (Fig. 1E). It has a translucent margin while most internal tissue is accumulated in the center as a yellow mass, which corresponds to the area below the ancestrula where it will start to dissolve the substrate (Fig. 1E).

### Ancestrula and early colony formation

**Fig. 3:**
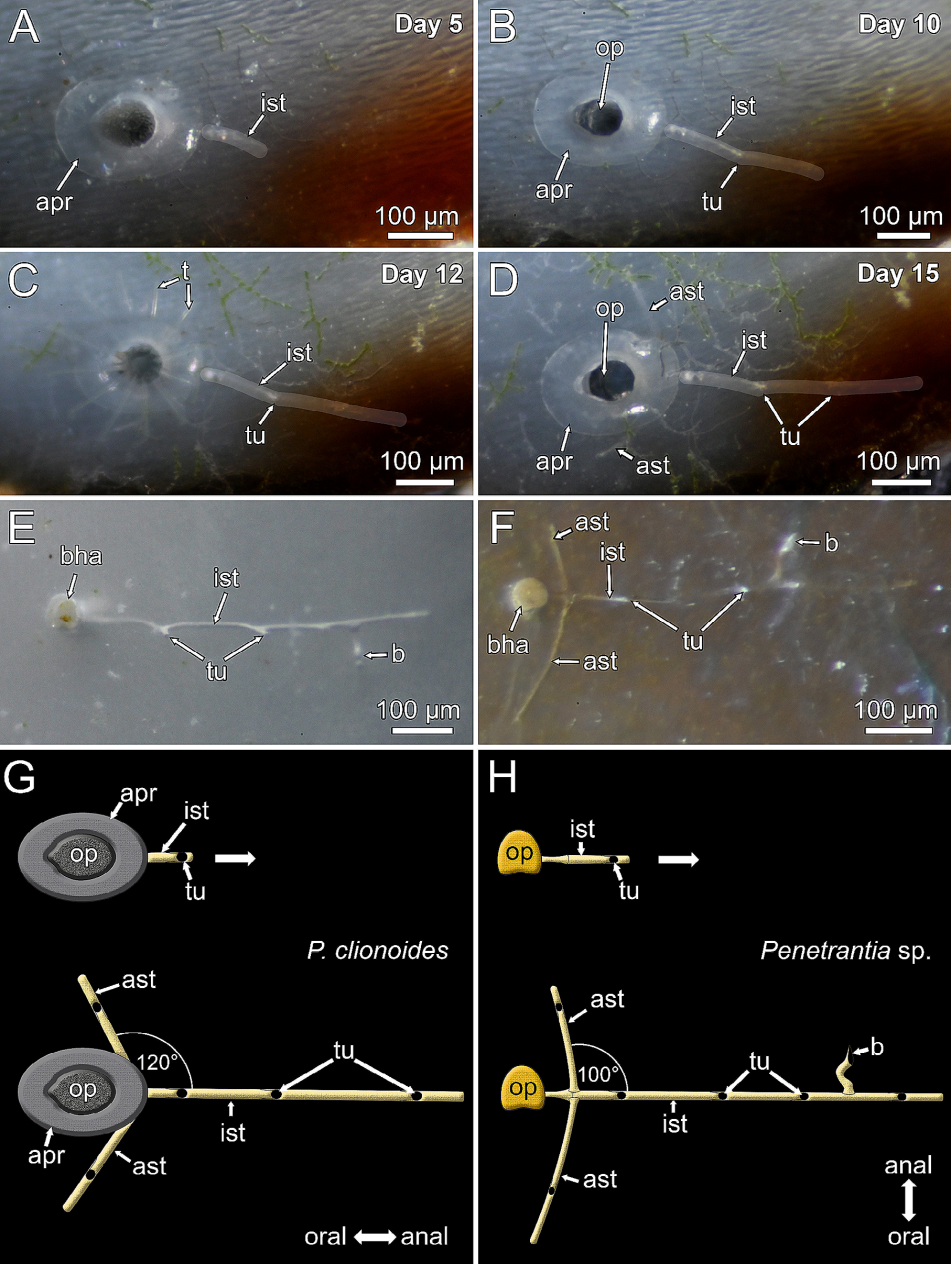
Stereomicroscopic images and schematic representation of ancestrula formation and colonial growth in *Penetrantia clionoides* from Guam and *Penetrantia* sp. from Roscoff, France. **A–D** Observation of ancestrulae of *P. clionoides* with intervals of days between images indicated. **C** On day 12 ancestrula protruded its lophophore for the first time. **E–F** Two different ancestrulae of *Penetrantia* sp. **G–H** Schematic representation of ancestrula–stolon complexes in *P. clionoides* and *Penetrantia* sp. respectively. *Abbreviations* apr – apertural rim, ast – additional stolon; b – bud; bha – bore hole aperture, ist – initial stolon, op – operculum, t- tentacle, tu - tubulet

All ancestrulae in *P. clionoides* and *P. japonica* had distinct apertural rims surrounding the borehole apertures. The rims were present only in ancestrulae and not in any other zooids (Figs. 2E and 3A–D). These rims were partially composed of calcium carbonate (Files S2, S3).

Actual ancestrula formation and metamorphosis were observed for three ancestrulae of *P. clionoides* only. After establishment of the preancestrula, it took 10 days until the aperture broke through, creating a hole in the center, while the rest of the cuticle around the borehole aperture formed an elliptical donut-shaped apertural rim, about 70 μm wide (Figs. 2E and 3A-D and F). There is a gap between the apertural rim and the substrate in *P. clionoides* and *P. japonica*, which becomes more evident when the apertural rim is eroded in older ancestrulae (Figs. 2D and E and 3A, B and D). After 12 days the operculum started to move and eventually the lophophore was able to protrude (Fig. 3C, Movie 1). Within these 12 days, the preancestrula transformed into a mature ancestrula and bored itself into the substrate approximately 300 μm deep (Fig. 3A–C). Simultaneously, the peduncle and the initial stolon developed and were already 300 μm long at the time of first lophophore protrusion (Fig. 3C).

Such detailed information about larval settlement and metamorphosis are missing for *Penetrantia* sp. from France as this species was not spawning in August to October 2022 nor in March 2023. However, two established ancestrulae were encountered in March 2023 (Fig. 3E, F). Ancestrulae in *Penetrantia* sp. lack apertural rims and do not differ from other zooids in external characters (Fig. 3E, F, H).

The orientation of the ancestrula to its initial principal stolon differs in *P. clionoides* and *Penetrantia* sp. (Fig. 3G, H). The peduncle forming the initial stolon developed on the anal side of *P. clionoides* ancestrulae (Fig. 3G) but on the lateral side in *Penetrantia* sp. (Fig. 3H). The initial stolon grew about 300 μm in *P. clionoides* (n3) (Fig. 3D, G) and 600 μm in *Penetrantia* sp. (n2) (Fig. 3F, H), before two additional stolons emerged on both lateral sides of their initial stolons. These three ancestrular stolons create a triradiate arrangement, radiating in different directions giving the ancestrula complex a distinct appearance (Fig. 3D, F, G, H). The angle between the central initial stolon and the lateral ones was about 120° in *P. clionoides* (n3) and 100° in *Penetrantia* sp. (n2) (Fig. 3G, H). In the latter species the first bud started to develop on the initial stolon before the additional lateral stolons emerged (Fig. 3E, H), while in *P. clionoides* the first pair of additional stolons developed before the first bud (Fig. 3D, G).

### Colonial growth and growth rates

**Fig. 4:**
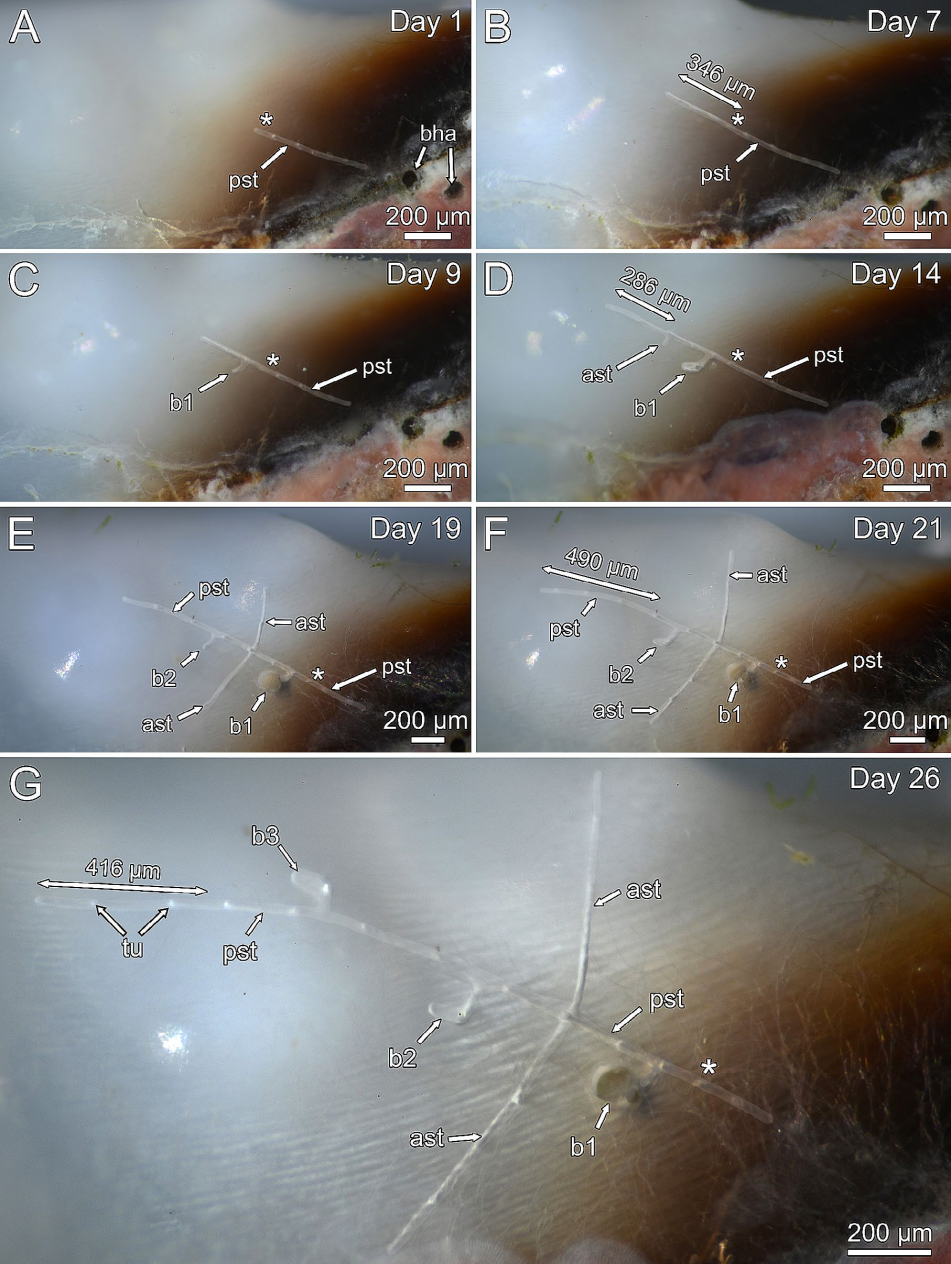
Colony growth of *Penetrantia clionoides* within 26 days. In its growth, the principal stolon either produces further autozooids or pairs of additional stolons in regular intervals on its lateral sides. **A** Beginning of growth experiment. Asterisk marks the length of principal stolon at the beginning of growth experiment. **B** Stolon extension within week one. **C** First bud emerges. **D** Stolon extension within week two and lateral stolon branch emerges. **E** Second bud emerges. **F** Stolon extension within week three. **G** Final size of colony which is reconstructed in Fig. 5. *Abbreviations* ast – additional stolon, b1 – bud1; b2 – bud2, b3 – bud3, bha – borehole apertures, pst – principal stolon, tu – tubulet

**Fig. 5:**
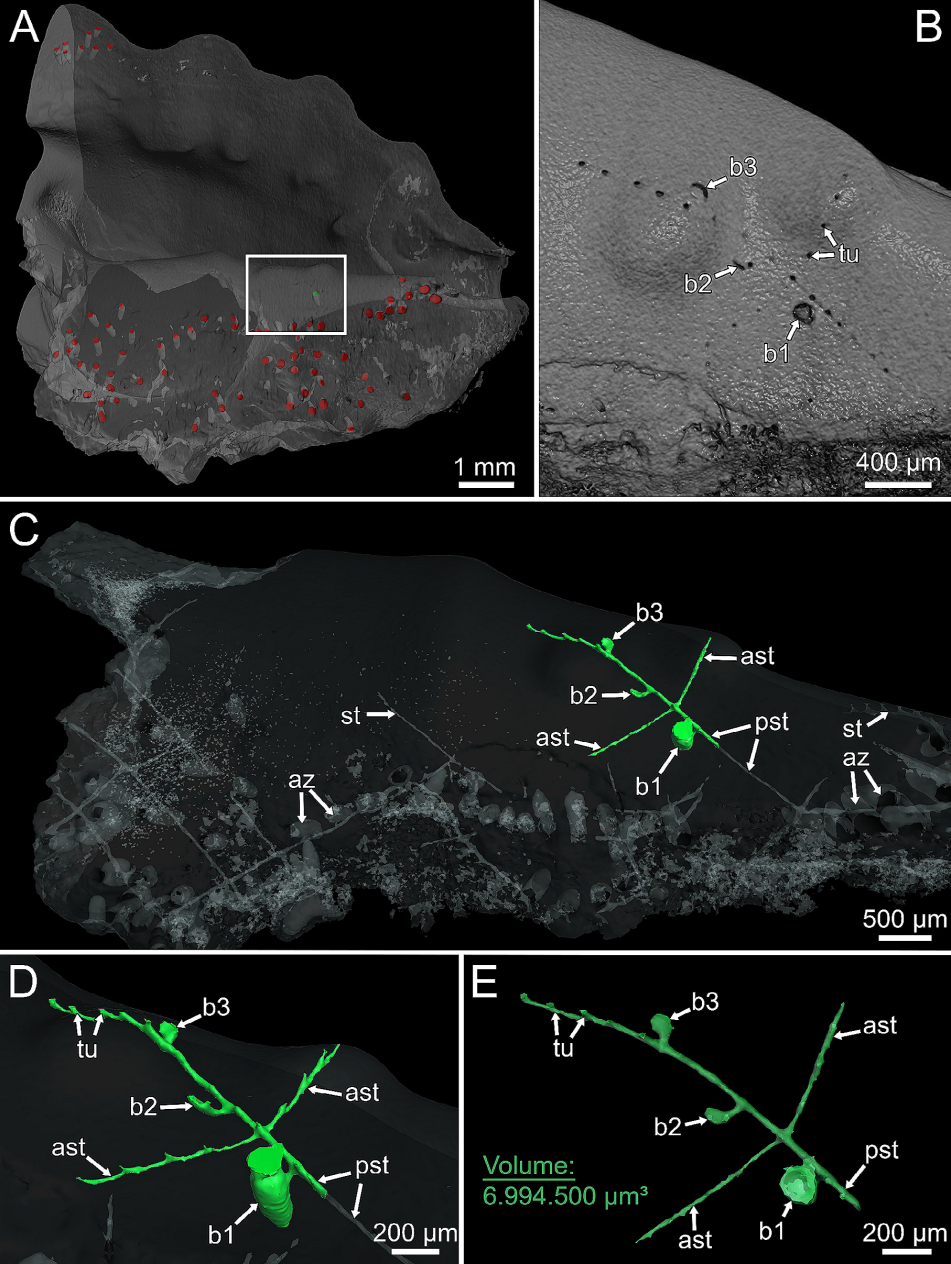
Micro-CT renderings of shell piece that contains the colony of *P. clionoides* observed during the growth experiment shown in Fig. 4. **A** Overview of bored shell of the gastropod *Drupa morum* with zooids labeled in red and bud1 in green. Rectangle indicates area of detailed scans shown in B–E. **B** Close-up of shell surface with boring traces of tubulets and apertures. **C–E** The colony part that developed during the growth experiment was segmented separately and is marked in green. The volume of the green colony part was calculated with the label analysis tool within the reconstruction software Amira. *Abbreviations* ast – additional stolon, az – autozooid, b1 – bud1; b2 – bud2, b3 – bud3, pst – principal stolon, st – stolon, tu – tubulet

Stolonal growth in *P. clionoides* and *Penetrantia* sp. followed the same pattern. While the principal stolon continued to grow in length, it produced either one autozooidal bud or a pair of additional stolon branches on its lateral sides, at consistent intervals of about 200 μm (Figs. 4 and 5C-E). The position of subsequent autozooids mostly alternates between the lateral sides of the principal stolon (left and right) but not regularly (Figs. 4C, E and G and 5C–D). Stolon branches always developed simultaneously on both lateral sides of the principal stolon (Figs. 4D–G and 5C–E). The development of autozooids followed the same pattern in both species and started with a lateral extension of the principal stolon. This extension then further progressed and bent about 90° in the frontal direction until it reached the surface of the substrate (Figs. 4C and D and 5D and E). Once the cystid reached the surface, the bud elongated further downwards in a basal direction until it reached its final size (e.g., bud1 in Figs. 4E and F and 5D and E). At the same time the future borehole aperture began to break through the surface (Figs. 2F, 4E and 5B). The entire developmental sequence into a mature autozooid took 13 days in *P. clionoides* and 31 days in *Penetrantia* sp., from the first bud anlage to the first protrusion of the lophophore (Figs. 4 and 5C–E; Movie 2, 3).

**Fig. 6:**
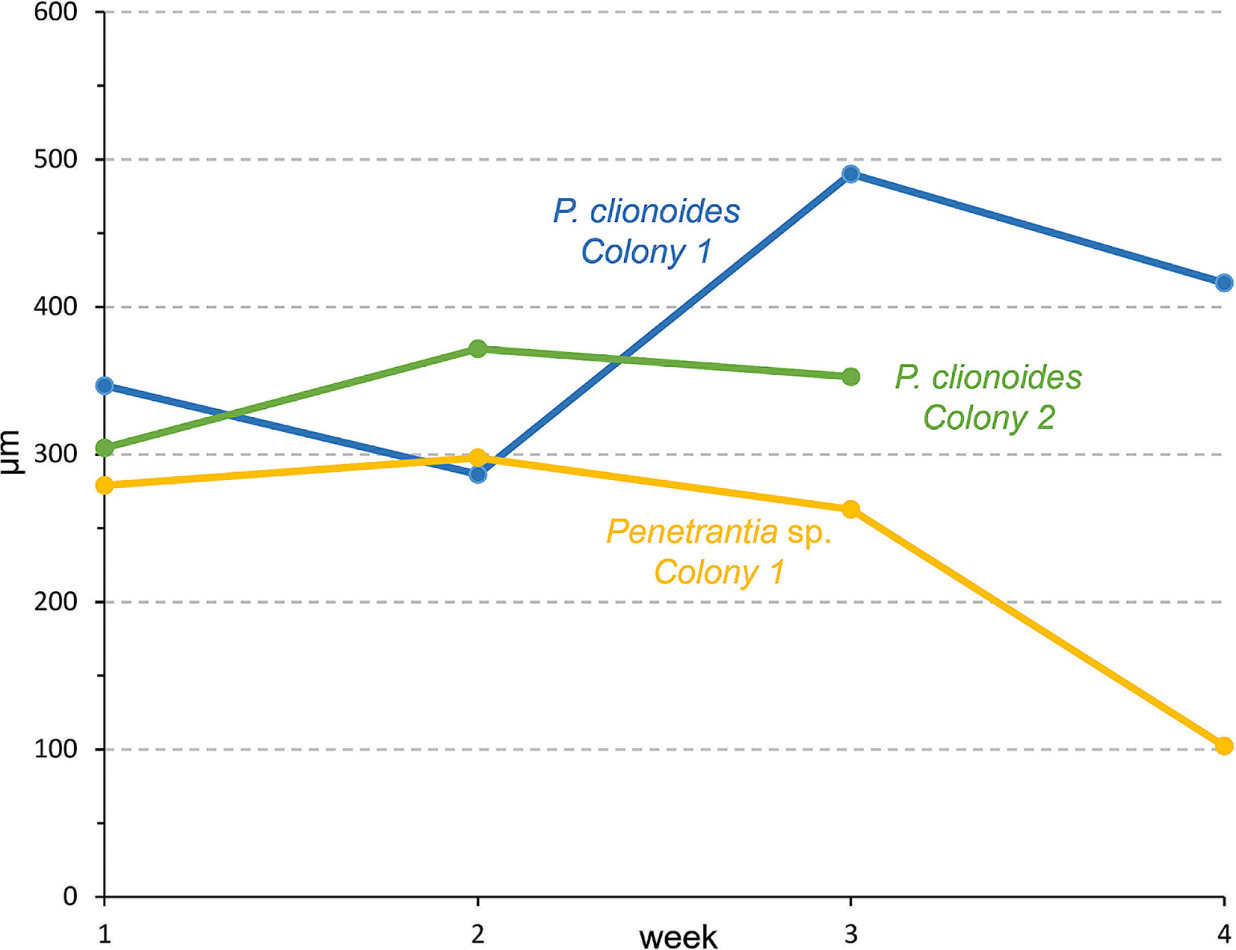
Weekly growth (length extension) of the principal stolons in three distinct colonies of two different penetrantiid species over a four-week period, based on measurements presented in Table 1. Including *Colony 1* (blue) and *Colony 2* (green) of *Penetrantia clionoides* from Guam, and *Colony 1* (yellow) of *Penetrantia* sp. from France

Likewise, stolonal growth was generally faster in *P. clionoides* than in *Penetrantia* sp. In two mature colonies of *P. clionoides* the overall mean of stolonal growth within four weeks was 335.2 ± 64.3 μm/week, *n* = 14 (Table 1; Figs. 4 and 5C–E). However, in *Colony 1* the mean growth of the principal stolon was more than 100 μm higher than of the additional lateral stolons (Table 1). In *Colony 2* the difference in the mean growth rates between principal and additional stolons is less distinct but in the principal stolon it was 24.7 μm higher per week (Table 1). The growth rates of individual stolons exhibited fluctuations between weeks. Overall, growth of the principal stolon in *Colony 1* of *P. clionoides* was the highest in week three (490.2 μm) and the lowest in week two (286.5 μm). In *Colony 2* of *P. clionoides* growth of the principal stolon was the highest in week two (371.6 μm) and the lowest in week one (304.3 μm) (Table 1; Fig. 6). The mean growth rate of the initial stolon of three ancestrulae in *P. clionoides* was 201.3 ± 132.7 μm/week, *n* = 6. In two ancestrulae the growth of the initial stolon was much lower in the second week than in the first week (Table 1; Fig. 3A–D).

**Table 1:**
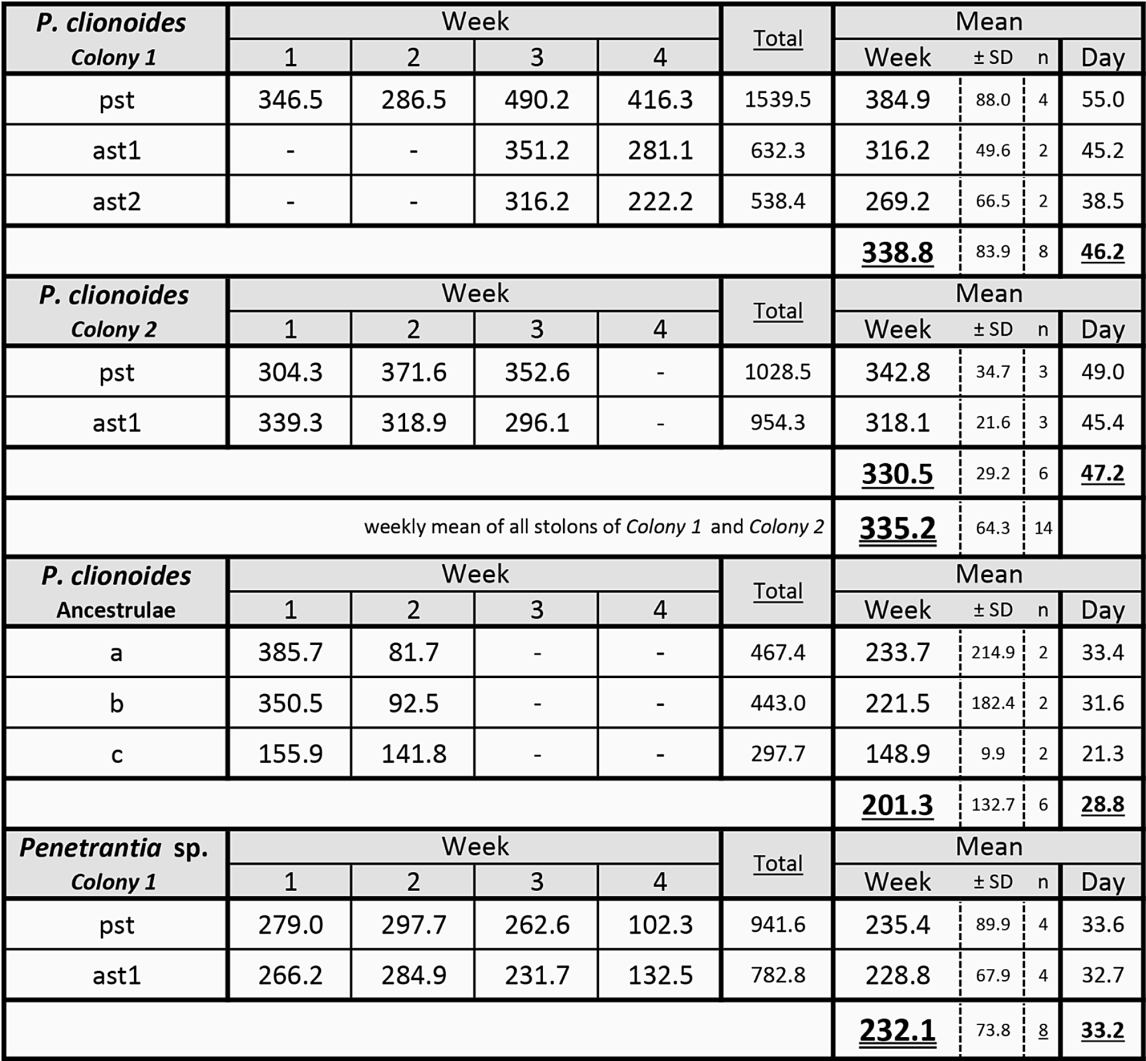
Colony growth rates of *Penetrantia clionoides* from Guam and *Penetrantia* sp. from Roscoff, France. Growth rates measured in length (µm) of stolon extension. Growth rates of ancestrulae correspond to the extension of their initial stolon

In a single colony of *Penetrantia* sp. the overall mean stolonal growth rate within four weeks was 232.1 ± 73.8 μm/week, *n* = 8, and therefore over 100 μm less than in *P. clionoides* (Table 1). Overall, the growth rate of the principal stolon in *Colony 1* of *Penetrantia* sp. was the highest in week two (297.7 μm) and about three times lower in week four (102.3 μm) (Table 1; Fig. 6).

In total, the observed colony of *P. clionoides* (Figs. 4 and 5) dissolved a shell volume of 6,994,500 μm³ within 26 days, which equals 0.0197 mg of aragonite or 0.0188 mg of calcite (aragonite density of 2.83 g/cm³, calcite density of 2.71 g/cm³, according to [25]). This translates to 1,883,134 μm³ per week (0.0053 mg aragonite, 0.0051 mg calcite) and 269,019 μm³ per day (0.00076 mg aragonite, 0.00072 mg calcite).

## Discussion

### Reproductive patterns

Bryozoans demonstrate a large variety of life-histories and reproduction patterns often with seasonal adaptations, correlating with their geographic and bathymetric distribution as well as with their colonial growth and larval type [3, 16, 26, 27]. Penetrantiids are no exception, and the presence of brooding gonozooids with embryos gives valid information about potential seasonal patterns in their reproduction [3, 11]. The number of gonozooids as well as incubating embryos varied drastically among colonies of *Penetrantia clionoides* from Guam and *Penetrantia* sp. from France, with *P. clionoides* demonstrating a much higher larval production overall. There are many reasons that can potentially alter reproductive patterns often associated with adaptations to seasonal changes in environmental conditions, e.g., temperature, food availability or substrate availability [3, 16, 20, 23]. *Penetrantia clionoides* and *Penetrantia* sp. live in very different environments offering an interesting comparison. While *P. clionoides* thrives in tropical intertidal habitats along the coast of Guam, *Penetrantia* sp. was found in the shallow subtidal zone of the temperate North Atlantic and North Sea [11]. Colonial organisms living in such temperate climate regimes usually show a stronger seasonality of asexual colonial growth and sexual reproduction, which strongly correlates with food availability [3, 20, 24, 26]. The cheilostome *Chartella papyracea*, similar to the penetrantiids, is a non-placental brooder that also occurs in the North Atlantic. Observations demonstrated that *C. papyracea* releases most larvae from autumn to spring, after the summer months when plankton concentration is highest and the colonies have enough nutrition to form gametes and larvae [26]. Since most young ancestrulae of *Penetrantia* sp. were encountered in February, larval release may have occurred in December/January. However, in this case we would expect a higher abundance of gonozooids in colonies from December. Perhaps, *Penetrantia* sp. is not seasonal and produces larvae, in smaller quantities, but all-year-round instead. In temperate climates, sexual reproduction tends to correlate with colonial growth: species with long-lived colonies (more than one year) tend to produce larvae throughout the year, whereas species with short-lived colonies often show distinct peaks in their reproductive cycle [3, 28]. The latter trend is also present in shallower intertidal species that show more distinct seasonality of growth and reproduction compared to species in deeper waters [3, 20, 28]. *Penetrantia* sp. may follow the reproductive strategy of long-lived colonies with a deeper bathymetric distribution, which is also underlined by lower growth rates than *P. clionoides* (see below).

Colonies of *P. clionoides* were only investigated in November and December and showed a high abundance of brooding gonozooids. Tropical species tend to have less distinct reproductive periods and lower reproductive rates stretched over the entire year [3, 29]. The exceptionally high number of brooding gonozooids in November could either represent a reproductive peak or *P. clionoides* may retain a high larval production throughout the year. Gonozooids of *P. clionoides* are able to brood larvae sequentially [11], which renders a longer reproductive phase plausible but also implies a high constant food uptake [3]. Guam has minimal seasonal changes in seawater temperature, but a distinct dry and wet season with large differences in rainfall [30]. The rainy season lasts from July to November [30], which places our observations on reproduction of *P. clionoides* at the end of this period. Observations on reproduction in the intertidal oyster *Saccostrea cucullata* from Guam showed a pattern with constant production of larvae throughout the year but with three distinct peaks, including one in November [31]. Such a reproductive pattern seems likely for *P. clionoides*, but its validation would require perennial observations. Nonetheless, the high accumulation of brown bodies in autozooids of colonies investigated herein indicates that they passed through several polypide regeneration cycles and probably were several months old [11, 32]. Brown bodies are encapsulated residuals resulting from polypide degeneration and either accumulate within a zooid, roughly indicating the number of polypide regeneration cycles, or are ejected by the subsequent polypide [32, 33]. Interestingly, no brown bodies have been observed in penetrantiid gonozooids, suggesting either that these gonozooids are younger than autozooids or that they do not generate brown bodies at all. Since brown bodies have never been observed in any penetrantiid gonozooid [11, 14], polypide regeneration might not take place in these heterozooids and as a consequence brown bodies do not accumulate in gonozooids [11]. Penetrantiid gonozooids are not capable of feeding since they have reduced polypides, lacking a digestive tract and in most cases a lophophore, which renders the production of brown bodies unnecessary [3, 11, 14, 32]. However, some bryozoans are capable of expelling or ejecting brown bodies after a polypide degeneration event, but since such a mechanism has never been observed in any penetrantiid and their autozooids commonly have many brown bodies incorporated, such an ejection mechanism is probably absent in penetrantiids [8, 11, 14].

Questions also remain regarding the formation and development of penetrantiid gonozooids. A complete developmental sequence has still not been documented, and in the current study, all investigated gonozooids were already fully developed. Gonozooids in different developmental stages have only been observed in *Penetrantia densa*, suggesting that the brood chamber develops early in the ontogeny of a gonozooid [14]. Since most gonozooids are also noticeably shorter than their corresponding autozooids, they seem to develop independently from autozooids and thereby represent true polymorphic heterozooids [3, 11]. Unlike many ovicells in cheilostomes, the brood chamber of penetrantiid gonozooids does not represent a kenozooid, but rather an outgrowth of the gonozooidal body wall [11, 16, 34]. The entire gonozooid, including its brood chamber, is lined by one continuous body wall and only the brood chamber plug separates the brood chamber from the gonozooidal tube, with no pore plates between [11]. Consequently, the brood chamber represents an outgrowth of the gonozooid and not an individual kenozooid [11, 15, 16, 34]. Altogether, the brood chamber in penetrantiids is not homologous to cheilostome ovicells and represents another example of convergent evolution in this phylum [11, 16, 34].

### Larval settlement in *P. clionoides*

Lecithotrophic bryozoan larvae have a short free-swimming phase of only a few hours to a few days to find a suitable substrate for the future colony [3, 16, 17]. Identification of ancestrulae in *P. clionoides*, *Penetrantia japonica* and *Penetrantia bellardiellae* (see below, apertural rims) is a very useful tool to asses recruitment patterns of penetrantiid larvae. Settlement experiments with *P. clionoides* demonstrated that the free-swimming phase of its larvae is very short, maybe only a few hours or minutes, and might explain why so many ancestrulae are found in such close proximity to each other and their parent colony. Ideally, larvae form ancestrulae on uncolonized substrates, with no or little competition for space [3, 17]. Settling on the same substrate as the parent colony might ensure suitable substrate conditions, but will lead to over-colonization, intergrowth of colonies, and in case of endolithic bryozoans, probably to a faster deterioration of the substrate. Bryozoan larvae are known to follow environmental cues such as light conditions, gravity, temperature or chemical composition of the substrate [3, 35, 36]. After a free-swimming phase, larvae will perform an exploratory behavior, investigating chemical and mechanical properties of the surface of the substrate and its microbial film [3, 17, 37, 38]. Perhaps the presence of conspecifics triggers settlement in *P. clionoides* and *P. japonica*? Such philopatric settlement behavior is known from many bryozoan species and many other sessile colonial animals with short-lived larvae [1, 2]. This strategy might facilitate finding the same or similar environmental conditions, increase colonization rates and presumably also the chance of potential future breeding events but with the added cost of inbreeding [1, 3]. Aggregated settlement of larvae was also documented in the ctenostome *Alcyonidium hirsutum* [39], and sibling larvae of the cheilostome *Bugula neritina* are also known to settle in clusters close together; however, it is not clear if this is due to true kin-recognition or shared settlement preferences of the larvae [40]. We observed that *P. clionoides* brooded many larvae simultaneously, which could lead to mass-releases of larvae and if the substrate of the parent colony has enough space, larvae may settle immediately after release.

Larvae of *P. japonica* showed a strong tendency to settle on or in the sutures of dead gastropod shells occupied (hermited) by hermit crabs. These sutures are slightly grooved and may offer different hydrodynamic characteristics than the remaining shell, which may promote larval settlement and/or increases the survival rate of larvae settled in the sutures in contrast to other shell areas. Similarly, images of *P. bellardiellae* indicate that most ancestrulae were situated in the sutures of its gastropod substrate (see supporting information S1a and S4 in [41]), suggesting that this preference is common among penetrantiid larvae. Similar larval settlement preferences were observed in the ctenostome *Alcyonidium hirsutum*, which showed a strong tendency to settle in concavities of its algal substrate [39, 42]. In contrast, ancestrulae of *P. clionoides* were only encountered close to the aperture of live *Drupa morum* shells, an area devoid of periostracum in live gastropods, which suggests that larval preferences of penetrantiids may correlate with the presence and/or condition of the periostracum [11]. The periostracum is a protective organic layer that covers most of the outer shell in mollusks [43]. The tendency to preferentially settle on shell areas lacking an intact periostracum was already suggested for most penetrantiids [9, 11, 14]. *Penetrantia clionoides* was reported to predominantly bore into gastropod shells that were already occupied by hermit crabs [44]. In older, dead, and hermited shells, the periostracum is usually more worn and degraded [9, 11], allowing penetrantiid larvae access to the calcareous layer more easily [11]. Likewise, *Penetrantia* sp. from France was found predominantly in dead bivalve shells, again in areas without or degraded periostracum [11]. Periostracum-free areas probably have different mechanical as well as microbial properties which initiate settlement of larvae. Altogether, penetrantiid larvae seem to have two major settlement preferences (1) older shell parts with little or no periostracum (2) close proximity to conspecifics (philopatry) and/or siblings.

Once larvae find a suitable place for settlement a connection between larva and substrate is established, a process that is similar in most investigated gymnolaemate bryozoans (e.g., *Amathia gracilis*, *Bugula neritina*) [3, 17]. This process starts with eversion of the larval internal sac which incorporates secretory glands and effectively glues the larva to the substrate [3, 45]. During this attachment process the larva flattens and becomes a preancestrula which also marks the onset of the “catastrophic” metamorphosis where all the larval organs are rearranged [3, 17]. The same holds true at least for *P. clionoides* in which the yellow area in the center of the preancestrula resembles the area where the boring process takes place during the expansion of the future cystid. The outer more translucent margin corresponds to the cystid epithelium similar to preancestrulae found in the cheilostome *Watersipora arcuata* [46]. However, further observations of preancestrulae and ancestrulae in different developmental stages are necessary to verify this hypothesis.

### Ancestrula and early colony formation

The formation of the preancestrula takes only a few minutes, whereas the development into a mature ancestrula takes several days to weeks depending on the species [17, 46]. In most gymnolaemate ancestrulae a functional polypide develops within 2–4 days [20]. In the epiphytic ctenostome *Pherusella minima*, ancestrula formation takes 7–10 days [47], and in the stolonate ctenostome *Amathia gracilis*, five days [48]. In *P. clionoides*, ancestrula formation took considerably longer (12 days), possibly a result of the boring process of penetrantiid ancestrulae.

The apertural rim is a useful external character to identify the ancestrula of a colony which can give important information about recruitment patterns of larvae and colony growth.

These peculiar rims around some borehole apertures have been reported previously in four species (*Penetrantia densa*, *P. clionoides*, *P. bellardiellae* and *P. japonica*) but have never been associated with ancestrulae [14, 41, 49, 50]. The apertural rim was mentioned in *P. clionoides* as a calcareous margin around some borehole apertures and suggested to pertain to zooids undergoing polypide regeneration, with sealing of their apertures (see Fig. 4 in [49]). This idea cannot be verified, since no autozooid of *P. clionoides* was observed to have an apertural rim in the current study, despite evidence of several polypide regeneration cycles, evidenced by the number of brown bodies [32]. A similar apertural rim was also reported in *P. bellardiellae* from Papua New Guinea [41]. Images of the aforementioned species (see supporting information S1, S2 and S3 in [41]) show a few zooids with apertural rims that look strikingly similar to the apertural rims found in *P. clionoides*. They have the same overall size and shape (donut-shaped), are only present around a few zooids, are partially composed of calcium carbonate, and there is a gap between apertural rim and substrate [41]. Consequently, we consider zooids with an apertural rim in *P. bellardiellae* as ancestrulae too. The two species also share other characters such as distinct opercular features: composed of calcium carbonate, and with a crescent-shaped rough patch [11, 41]. Recently, zooids of *P. japonica* were also reported to have apertural rims [50] and this study confirms that they are also an ancestrular feature. The fourth species with calcareous apertural rims is *P. densa* from South Africa. However, these rims represent a narrow margin between the borehole and the surrounding substrate and it is indicated that autozooids also have such rims (see Fig. 56 in [14]). Consequently, the apertural rim in *P. densa* probably is not an ancestrular feature. Nevertheless, reinvestigation of *P. densa* is required to support this notion.

The apertural rim may represent the former larval cuticle or the pallial epithelium of the preancestrula [46], since the actual ancestrular cystid is formed below the preancestrula. This was evident in a few ancestrulae of *P. clionoides* where the exterior cuticle of the cystid is clearly separated from the apertural rim. Additionally, preancestrulae already have the same size as the future apertural rim, while the formation of the ancestrular cystid is ongoing [3, 47]. The incorporation of calcium carbonate into the body wall is atypical for ctenostome bryozoans, but since, *P. clionoides*, *P. japonica* and *P. bellardiellae* also have calcified opercula, they might have the capability of biomineralization [11, 41]. However, whether true biomineralization or reuse and remolding of calcium carbonate released during the boring process occurs still needs to be validated. Nonetheless, the preancestrula/early ancestrula is already calcified shortly after settlement, indicating independent biomineralization. The reason why only some penetrantiids form apertural rims and others do not remains unknown, but we observed that *P. clionoides*, *P. japonica*, and *P. bellardiellae* occur exclusively in the intertidal zone and the calcification of their opercula might be an adaptation to this environment and potentially helps to better seal the aperture during low tide [41, 49, 50].

Another common feature of all investigated penetrantiid ancestrulae is the early stolonal pattern with three stolons radiating in different directions to create a triradiate arrangement [9, 11], whereas autozooids are commonly associated with a single stolon [9, 11, 14]. The peduncle and initial stolon develop simultaneously at the ancestrular zooid in all investigated penetrantiids, hence the term ancestrula–stolon complex, as the ancestrula effectively consists of two zooids: the feeding ancestrula and its first kenozooidal stolon. A similar pattern with the stolon forming already in the preancestrula was observed in the ctenostome *Amathia gracilis* [48]. The precise timing of stolon development in *P. clionoides* could not be determined, but the initial stolon was present before first lophophore protrusion. Although the overall outline is similar, there are differences between the ancestrula–stolon complexes in *P. clionoides* and *Penetrantia* sp. from France. Most obvious are the different budding sites of the peduncle and initial stolon (anal in *P. clionoides* and lateral in *Penetrantia* sp.) and the delayed development of the first pair of additional stolons in *Penetrantia* sp. However, in both species the first pair of additional stolons are not part of the ancestrula–stolon complex as they develop after the ancestrula and initial stolon are established. The angle between the central initial stolon and the first pair of additional stolons was consistent in all investigated specimens. Whether this serves as a reliable species character needs detailed investigations of other penetrantiids.

### Colonial growth and growth rates

The overall growth pattern of *P. clionoides* and *Penetrantia* sp. corresponds well to typical penetrantiid colony forms, where stolon branches and zooids develop on the lateral sides of a principal stolon, eventually leading to strongly ramified and feather-shaped colonies [9, 11, 14]. However, this pattern becomes complicated as penetrantiid ancestrulae were often observed in close proximity (see above), suggesting that one large ‘colony’ can probably be the result of several intertwined individual colonies.

Autozooid development is similar in *P. clionoides* and *Penetrantia* sp. and follows the same sequence as described for *P. densa* [14]. Consequently, this process seems to be uniform among Penetrantiidae. Gonozooids are considered to develop separately and independently from autozooids (see above) [11, 14].

Data on growth rates of bryozoans are generally rare and even more so for endolithic organisms, including boring bryozoans. Most bryozoan growth rates have been estimated for species with larger colonies and show great variations across different species but also between colonies of the same species from different localities [3, 51–53]. Allowing for the large diversity in growth forms and colony structures, there are many different ways to measure growth, e.g., colony diameter, branch length, surface area or zooid number, making direct comparisons ambiguous [51, 52, 54, 55]. In the case of encrusting cheilostomes, the linear extension of their colonies varies greatly across different species, with an estimated mean of 3.2 mm/year [52]. Growth data on ctenostome colonies is mostly restricted to a few epiphytic and encrusting species in temperate waters, e.g., *Alcyonidium hirsutum* and *Flustrellidra hispida* [21, 39, 56]. However, these growth rates can hardly be compared to stolonate ctenostomes, where colony growth is mostly reflected in proliferation and extension of their stolons while autozooid density is much lower [8, 57]. Consequently, we measured the change in stolon length in our analysis. The mean growth rate of stolons in *P. clionoides* (335.2 μm/week which translates to 18.3 mm/year) was much higher than the mean extension of encrusting cheilostome colonies (3.2 mm/year) [52]. However, stolon extension is expected to be faster than colony extension by autozooidal budding, since there is no polypide development involved in stolonal growth, highlighting the incomparability of these growth rates. Although stolon ontogeny and colony growth pattern have been documented for several stolonate ctenostomes, growth rates were not documented [8, 57, 58].

Distinct differences are present in stolonal growth and autozooid development between *P. clionoides* and *Penetrantia* sp. The tropical *P. clionoides* displayed much higher growth rates than the temperate *Penetrantia* sp., which most likely correlates to the warmer water temperatures in Guam (Guam: 28–30 °C Guam; France, Roscoff: 14–18 °C). Temperature, food availability, and colony size, are all known to have large effects on bryozoan growth rates, which is also reflected in lower growth rates of most bryozoans from higher latitudes [3, 20, 52, 59–61]. Food supply and availability may also be a major factor in our analysis. In both cases, the unfiltered-seawater was taken from the intertidal zone, but *Penetrantia* sp. inhabits the subtidal and therefore food composition might not have matched its species-specific preferences, though colonies were supplied with a culture mixture. These presumably suboptimal conditions might also be the reason why growth rates in *Penetrantia* sp. further decreased during the experiment, while *P. clionoides* showed more constant growth rates that even increased in the case of two stolon branches. Growth experiments on the cheilostome *Membranipora membranacea* showed significantly lower growth rates of colonies kept under laboratory conditions compared to colonies observed in the field, which indicates that reproducing optimal conditions in the laboratory remains difficult [60]. Growth rates in this species are also affected by colony size, with larger colonies growing exponentially faster [60]. This might explain why stolons of ancestrulae in *P. clionoides* had lower growth rates than mature colonies. Consequently, colonial growth rates in *P. clionoides* probably correlate with the number of feeding autozooids. However, many species are also known to reach a peak in growth at a certain colony size—or, in some cases, growth even decelerates [62]—often correlating with reproductive phases when colonies invest more energy into larval production [63]. Differences in growth rates may also be influenced by varying decalcification rates, attributed to the nature of substrates inhabited by *P. clionoides* and *Penetrantia* sp. While the tropical *P. clionoides* inhabits live gastropod shells, the temperate *Penetrantia* sp. inhabits deceased bivalve shells. This distinction suggests varying mineralogical compositions (calcite vs. aragonite) and organic content levels between the two substrates. There is a clear latitudinal gradient in molluscan shell mineralogy, with species from higher latitudes generally exhibiting a higher amount of calcite in their shells, while species from lower latitudes tend to have a higher amount of aragonite [64, 65]. Shells with a higher calcite content are typically more robust and less soluble [64], which could potentially explain the lower growth and decalcification rates observed in the temperate *Penetrantia* sp. However, bivalve shells from the Arctic Ocean were recently discovered to be primarily composed of aragonite, contradicting the previously observed latitudinal gradient [66]. Additionally, the bivalve shell was already deceased and more porous than the live tropical gastropod shell, which should have increased the growth rates of *Penetrantia* sp. Consequently, the shell composition of the temperate bivalve *Anomia ephippium*, as well as its older (dead) state, should have favored faster growth of *Penetrantia* sp. from France. However, the opposite was observed, suggesting that other environmental parameters such as temperature and food availability had a larger impact in this case. In general, substrate properties are expected to have a significant impact on the decalcification rates of boring bryozoans, especially between shells composed entirely of calcite versus those composed solely of aragonite.

We observed a similar trend in the duration of autozooid formation by asexual budding as for stolonal growth, which was faster in *P. clionoides* than in *Penetrantia* sp. (13 days vs. 31 days, respectively). These differences most likely correlate with the stolon growth rates and are underlined by the same environmental parameters as mentioned before (temperature, food availability, colony size, and shell mineralogy) [39, 56, 60, 61]. In general, asexual autozooid formation in both penetrantiid species seems to be slower than in most epibenthic bryozoans including other ctenostomes [21, 39, 52, 56]. This indicates that the endolithic lifestyle requires a longer developmental period of autozooids, as they have to chemically dissolve the substrate during their growth. Growth rates of other endolithic organisms are little documented and often restricted to larger macroborers (e.g. sponges, polychaetes) that mostly use a combination of chemical and mechanical mechanisms [19]. Bioerosion rates are about five times higher in the tropical boring sponge *Cliona orientalis* than in its temperate counterpart *Cliona celata*, indicating that bioerosion rates are higher in lower latitudes [67]. This could be another factor for *P. clionoides’* higher growth rates, occurring as it does at lower latitudes.

### Impact on bioerosion

The impact of boring bryozoans on bioerosion is unknown, since there are neither published growth experiments nor dedicated bioerosion experiments available. So far, the impact of penetrantiids on their substrate has been considered minimal as they penetrate only the superficial layers (upper 100–500 μm) of the substrate, and live gastropods were assumed to be unaffected by the boring activity [9, 11, 14]. Consequently, the relationship between boring bryozoans and their substrate is considered to be non-parasitic [9, 14].

All boring bryozoans are considered internal microborers according to their size (diameter < 100 μm) and the utilization of a chemical boring mechanism only [18, 19, 68, 69]. Nonetheless, since the true abundance, distribution and growth rates of penetrantiids has historically been largely underestimated, their impact in bioerosion is probably higher than previously estimated especially in tropical waters [11]. We have encountered shells that were completely bored by penetrantiids, which alters substrate properties and facilitates other bioerosion effects such as grazing [70, 71]. Additionally, most microborers play a key part in bioerosion processes and are considered pioneer borers that often start the penetration followed by small macroborers like polychaete worms, enabling subsequent larger macroborers to colonize the substrate [71, 72].

Herein, we provide the first estimations in bioerosion rates of a boring bryozoan species. Assuming the observed colony of *P. clionoides* has a linear growth rate throughout the year, it would dissolve 0.09 mm³ of its substrate within one year which translates to 0.2778 mg of aragonite or 0.2651 mg of calcite (according to [25]), However, the assumption of a linear growth rate is speculative since most bryozoan species exhibit seasonal fluctuations in their growth rates as their colonies become larger, with more growing edges and more feeding autozooids [60]. Consequently, the actual bioerosion rate of *P. clionoides* is probably higher. Nevertheless, our findings give the first indication of the extent to which penetrantiids contribute to bioerosion. However, it is not easy to compare these values since information on internal bioerosion is scarce, particularly for microborers. The boring sponge *Cliona celata* erodes 1.94–2.55 mg per day [67], which is much higher than our estimate for *P. clionoides* from Guam (0.0007 mg per day). But the sponges in the aforementioned experiment were larger and lived within even larger oyster shells than our *P. clionoides* colony, making this comparison biased. Future studies should conduct dedicated bioerosion experiments on boring bryozoans, including information on the eroded substrate area, to generate comparable data in kg m ^− 2^ year ^− 1^ [18, 19, 67].

## Conclusion

The life history of penetrantiids appears highly adapted to their endolithic lifestyle and their geographic, as well as bathymetric, distribution. The presence of brooding gonozooids with embryos offers insights into their reproduction patterns, with *P. clionoides* demonstrating notably higher sexual reproductivity. In contrast, *Penetrantia* sp. from temperate waters may exhibit less distinct reproductive peaks. This distinction is further emphasized by different colonial growth rates, with *P. clionoides* exhibiting notably higher growth rates, likely influenced by higher water temperatures and optimal food availability.

Larval recruitment patterns suggest philopatric behavior in both *P. clionoides* and *P. japonica*, potentially driven by conspecific cues or shared settlement preferences. Settlement on or near specific substrate features, such as sutures in gastropod shells, might optimize larval survival and colony establishment, with penetrantiid ancestrulae generally found in areas where the periostracum layer is absent or worn.

Ancestrula formation and early colony development revealed unique anatomical features, such as apertural rims and the triradiate stolon arrangement, providing valuable insights into penetrantiid taxonomy, settlement preferences, and colony formation. Although ancestrulae of all investigated penetrantiid species form star-shaped ancestrula–stolon complexes, there are species-specific differences that provide additional diagnostic characters, such as: (1) varying budding sites of the initial stolon, (2) differences in the timing of development in the first pair of additional lateral stolons, and (3) variations in the angles between initial and additional stolons.

The impact of penetrantiids on bioerosion remains poorly understood, although we have highlighted potential implications for substrate alteration and facilitation of other bioerosion processes. Additionally, we have generated first projections of bioerosion rates for the tropical species *P. clionoides*, along with a protocol for maintaining *Penetrantia* under laboratory conditions.

Overall, this study underscores the importance of understanding the intricate relationships between penetrantiid bryozoans and their environment, contributing to broader insights into marine ecosystem dynamics and bioerosion processes. Further research, including dedicated bioerosion experiments and long-term ecological monitoring, is essential to fully comprehend the ecological roles and evolutionary adaptations of these intriguing organisms.

### Electronic supplementary material

Below is the link to the electronic supplementary material.

Supplementary Material 1

Supplementary Material 2

Supplementary Material 3

Supplementary Material 4

Supplementary Material 5

Supplementary Material 6

Supplementary Material 7

## Data Availability

The datasets used and/or analyzed during the current study are available from the corresponding author on reasonable request.
