# Supplementary figures and images for: Boring life: early colony formation and growth in the endolithic bryozoan genus Penetrantia Silén, 1946
Source: Zoological Lett. 2024 Jun 14;10:10. doi: 10.1186/s40851-024-00234-z (PMC11179354; doi:10.1186/s40851-024-00234-z)

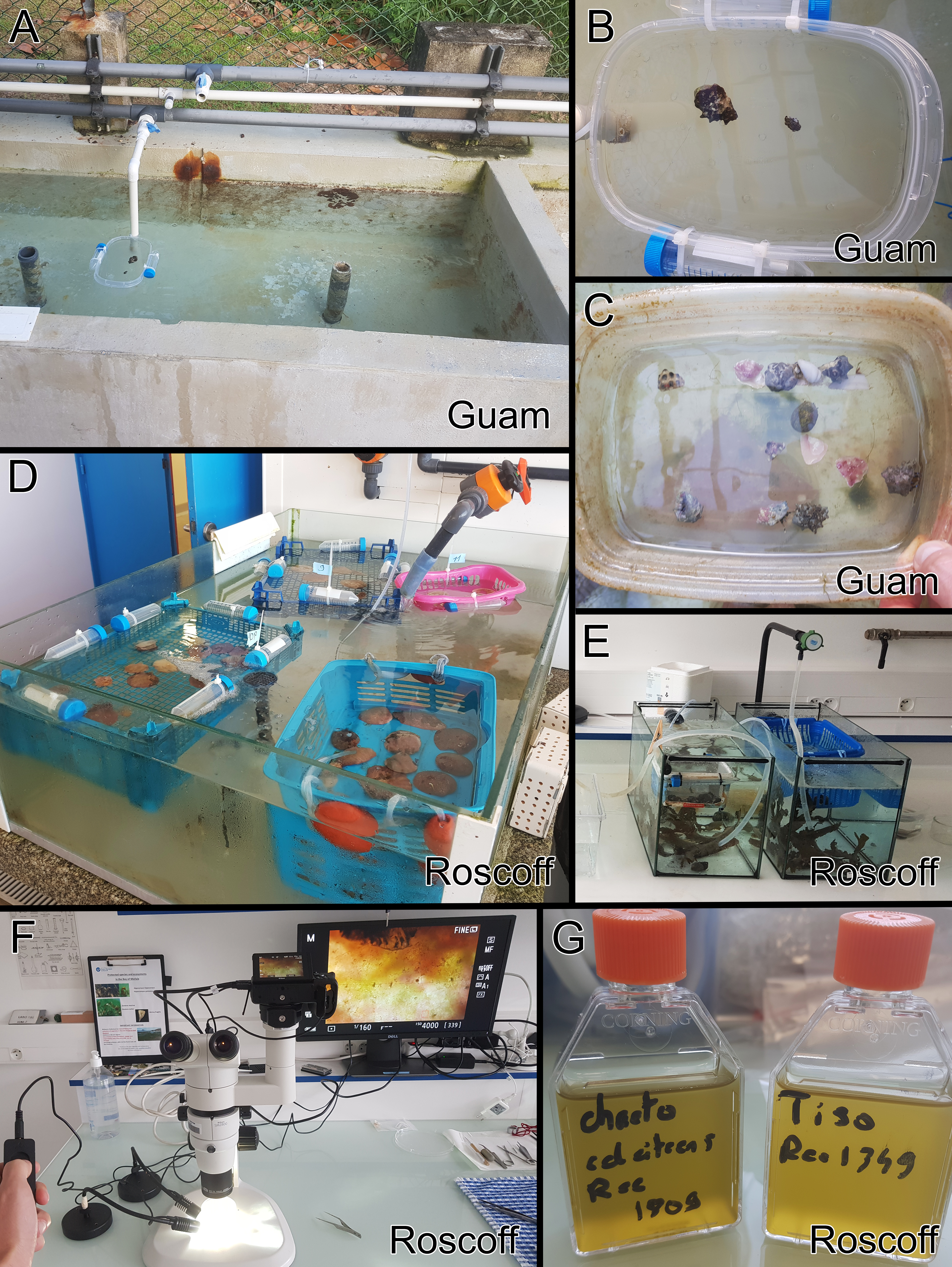

Supplement: Supplementary file 1 — Supplementary Material 1 [file 40851_2024_234_MOESM1_ESM.jpg]
