# Supplementary material for: Boring life: early colony formation and growth in the endolithic bryozoan genus Penetrantia Silén, 1946
Source: Zoological Lett. 2024 Jun 14;10:10. doi: 10.1186/s40851-024-00234-z (PMC11179354; doi:10.1186/s40851-024-00234-z)
Supplement: Supplementary file 2 — Supplementary Material 2 [file 40851_2024_234_MOESM2_ESM.pdf]

2023-09-21

Author:	CIUS EM - Lab
Creation:	09/21/2023 8:34:36 AM
Sample Name:	1

1

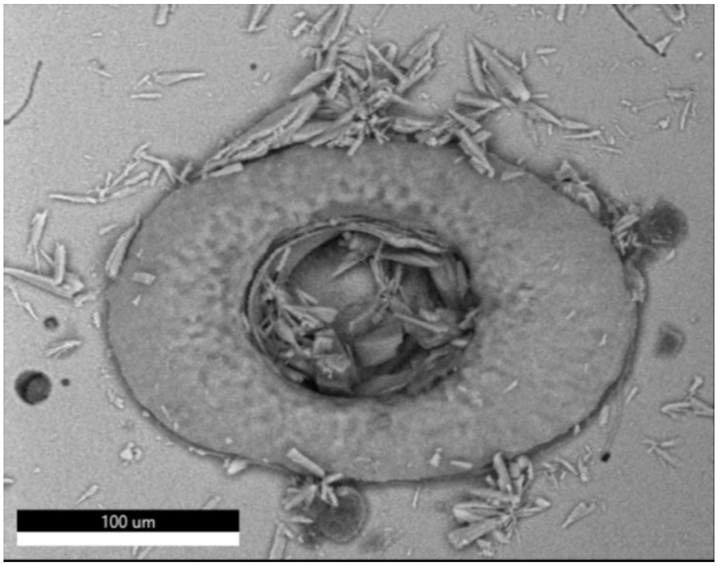

Image

Live Map 1

ElementOverlay

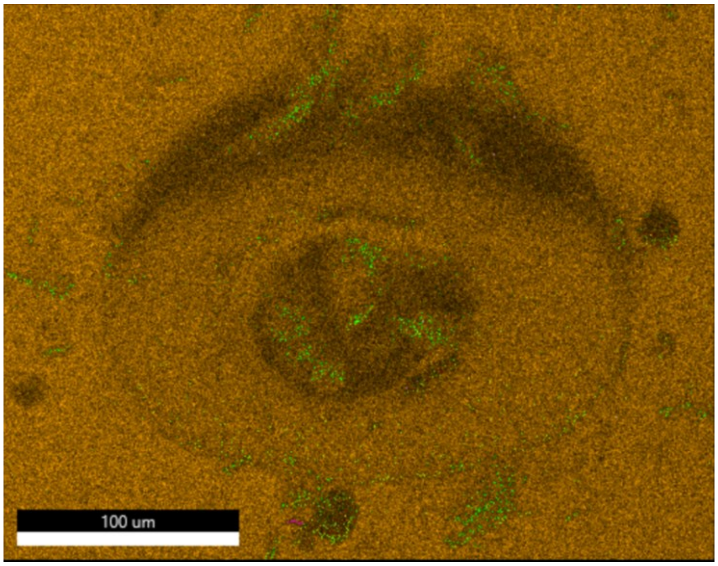

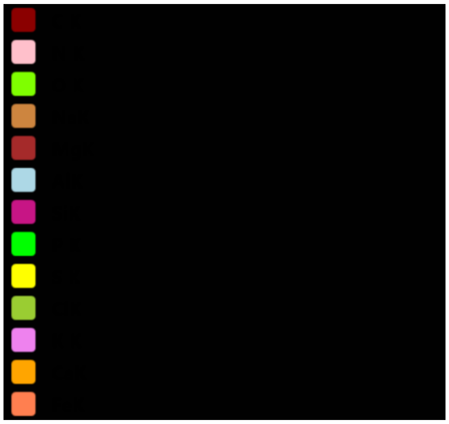

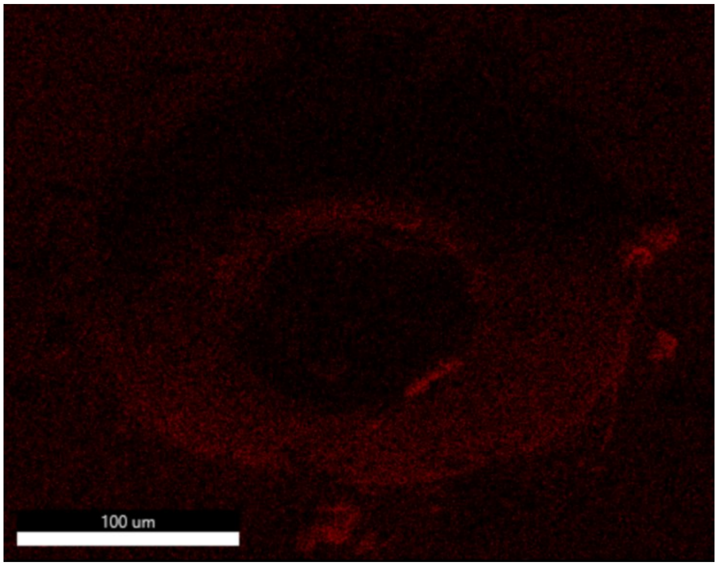

C K_ROI (11)

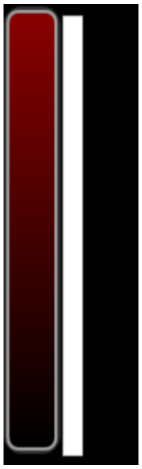

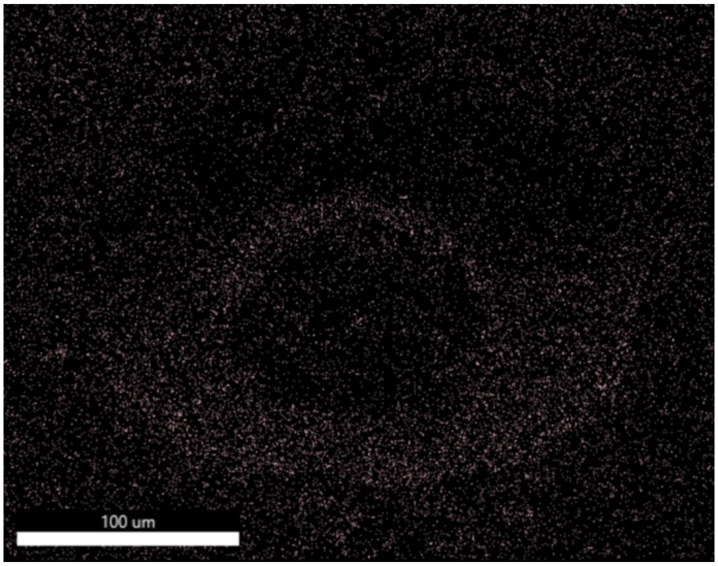

N K_ROI (4)

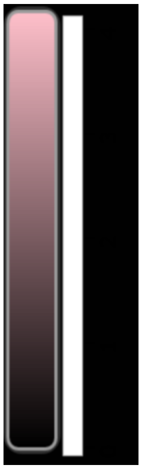

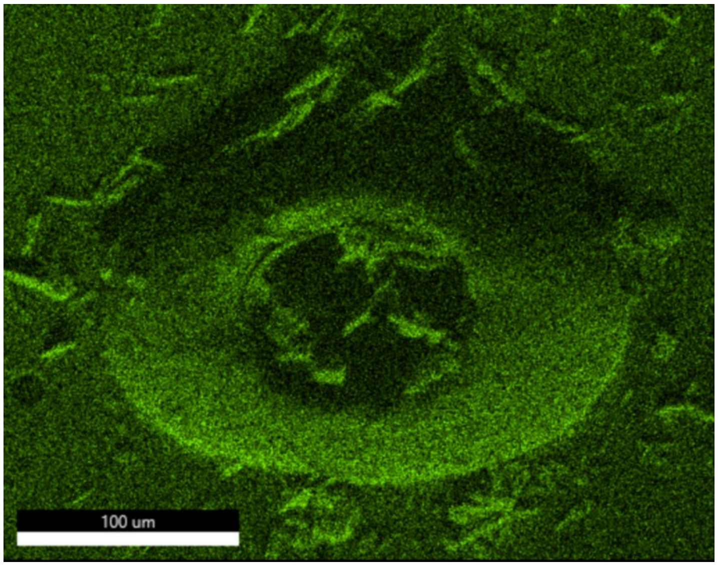

O K_ROI (18)

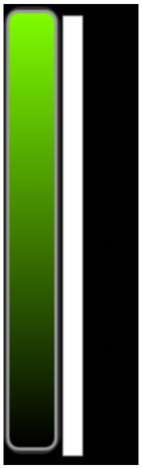

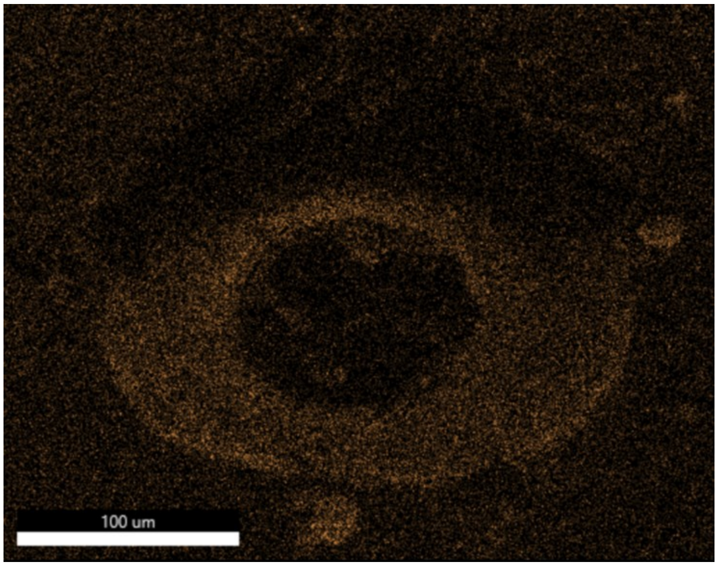

NaK_ROI (9)

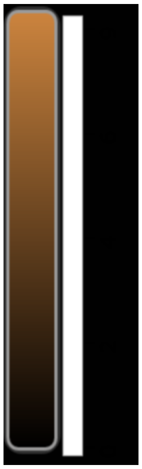

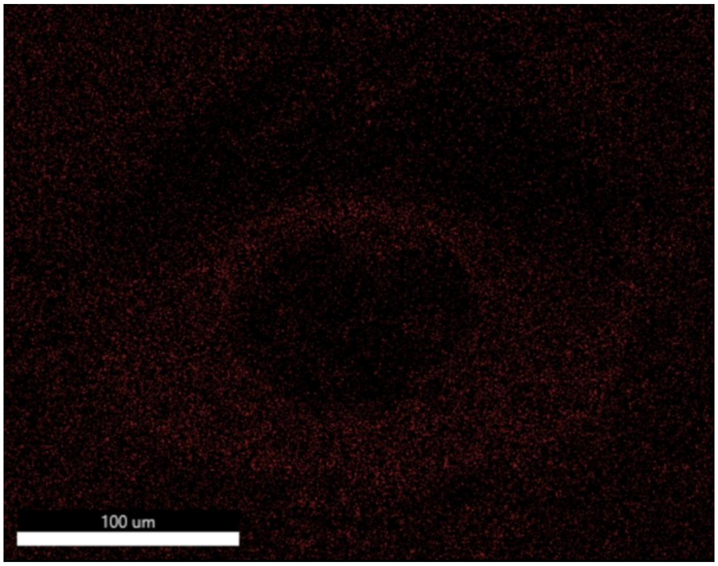

MgK_ROI (6)

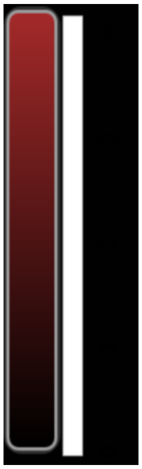

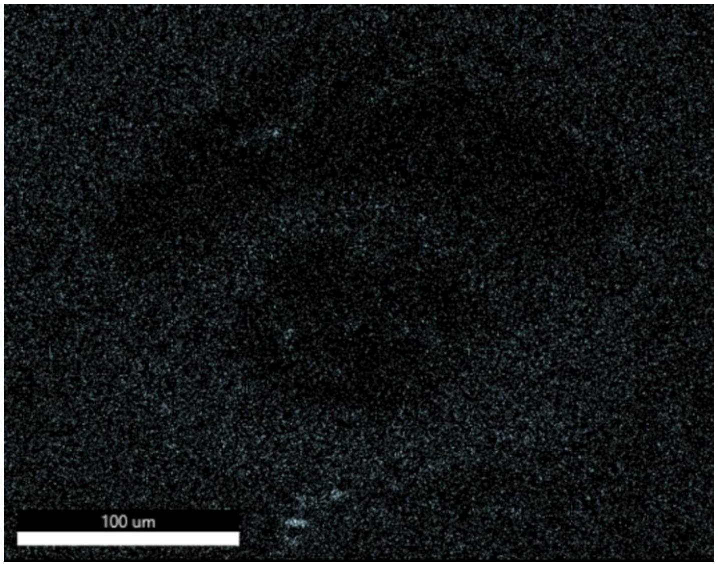

AlK_ROI (8)

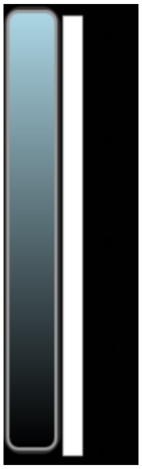

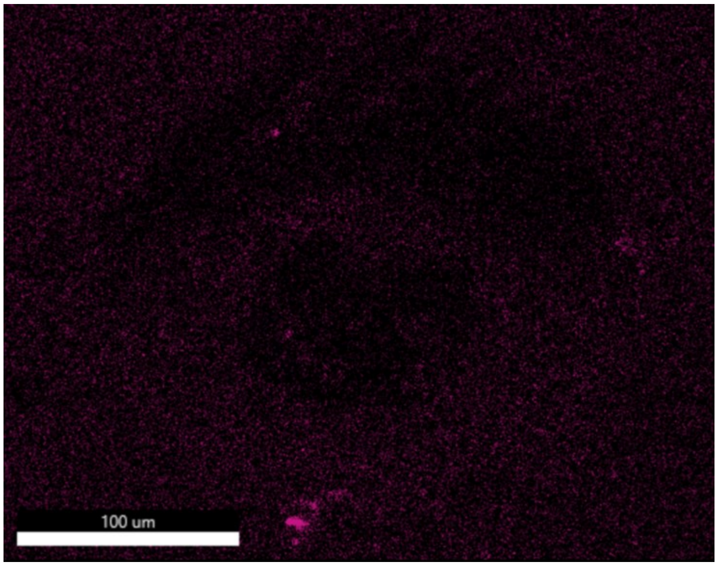

SiK_ROI (13)

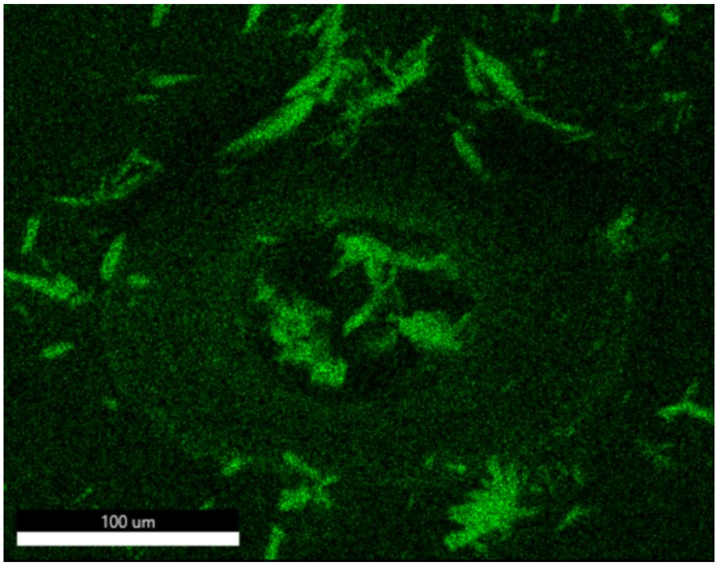

P K_ROI (21)

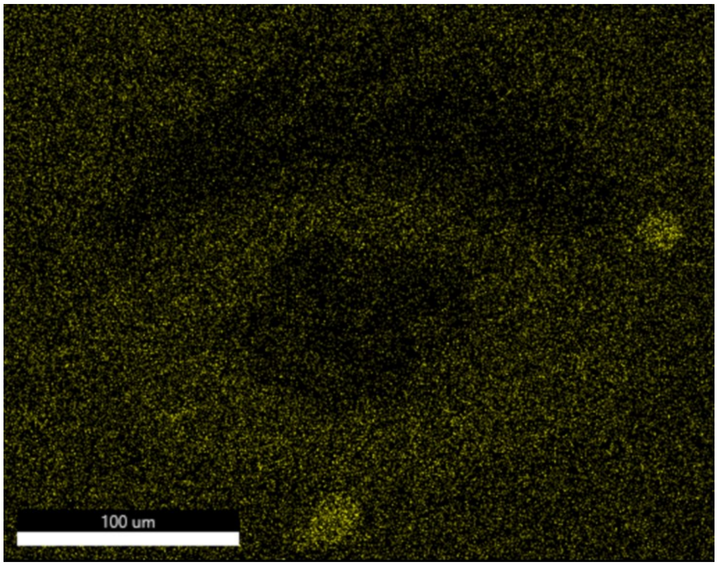

S K_ROI (7)

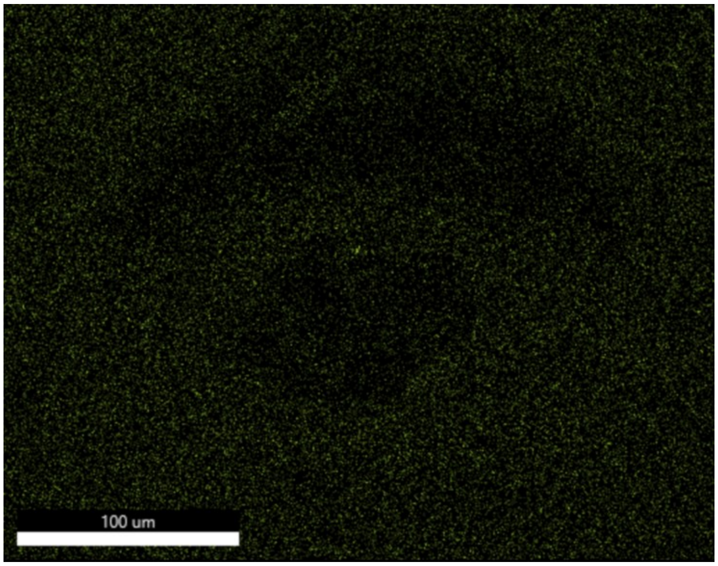

ClK_ROI (6)

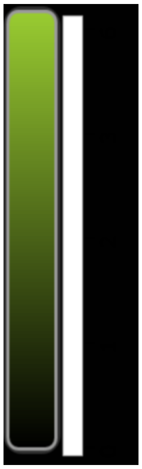

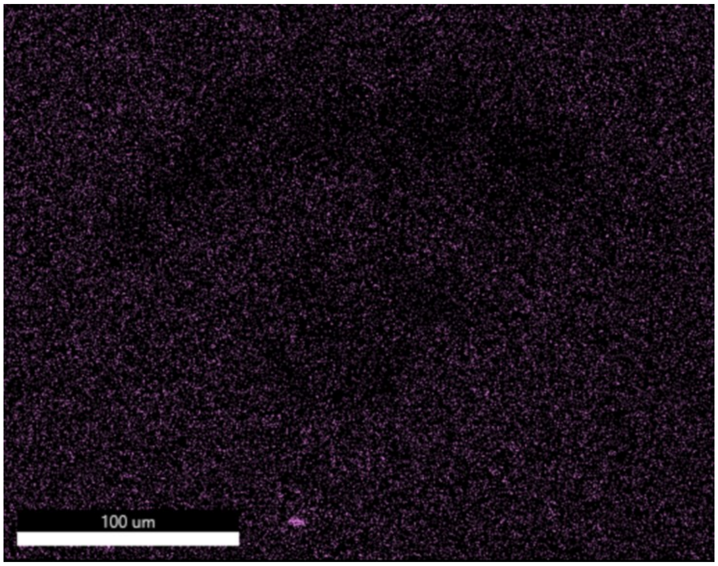

K K_ROI (9)

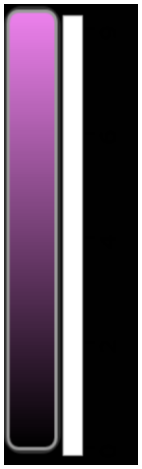

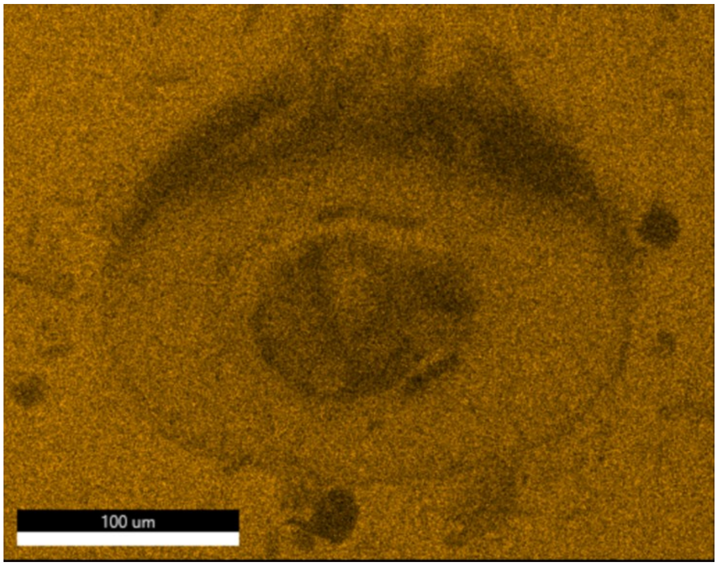

CaK_ROI (44)

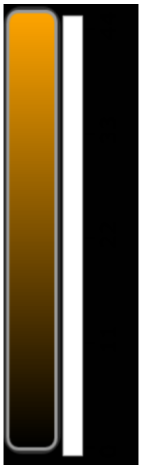

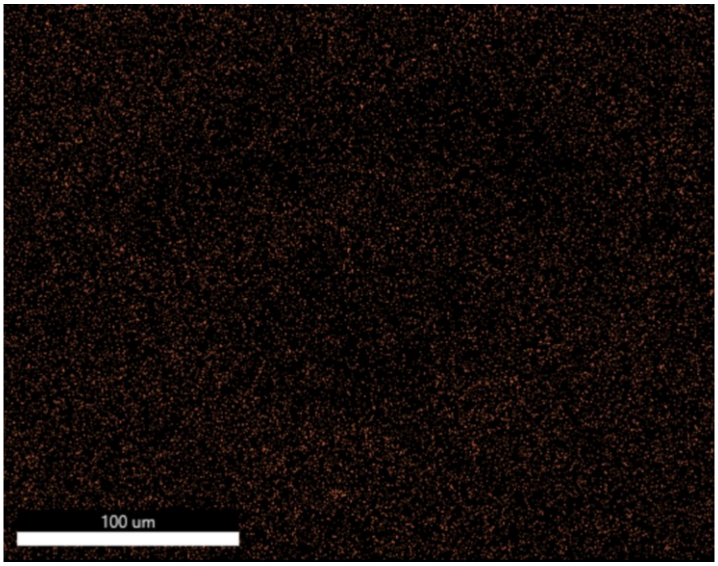

FeK_ROI (7)

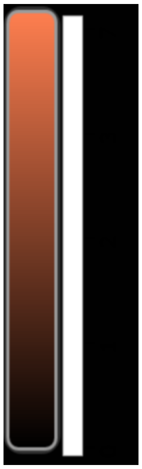

kV: 20 Mag:400 Takeoff: 35.6 Live Time(s): 655.4 Amp Time(µs): 7.68 Resolution:(eV)127.4

Sum Spectrum

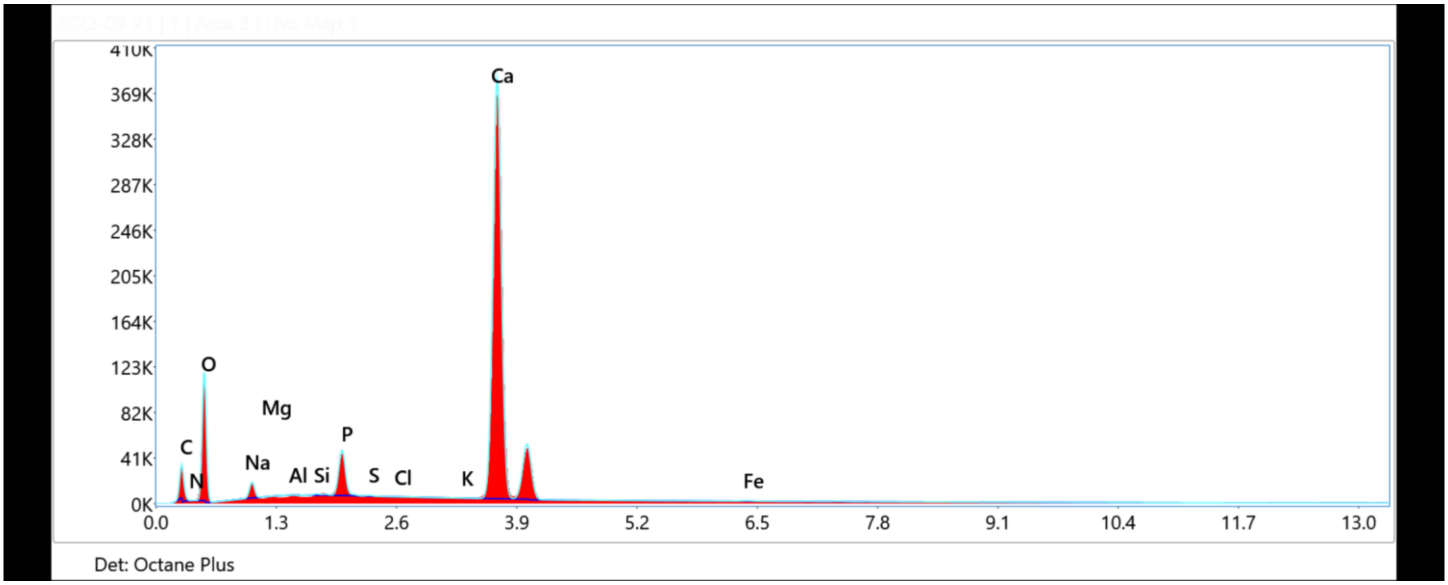

<u>Quant Results</u>

Element	Weight %	Auto MDL	Atomic %	Net Int.	Error %	R	A	F
C K	14.15	0.12	22.63	204.98	10.22	0.8956	0.1097	1.0000
O K	49.16	0.06	59.00	982.17	10.33	0.9069	0.0709	1.0000
Na K	1.49	0.02	1.25	145.93	9.34	0.9199	0.2165	1.0034
Si K	0.05	0.01	0.04	15.49	8.71	0.9311	0.6092	1.0186
P K	1.88	0.01	1.17	529.93	4.24	0.9344	0.7148	1.0284
S K	0.03	0.01	0.02	9.33	14.13	0.9376	0.7816	1.0456
Ca K	33.13	0.02	15.87	6336.41	1.84	0.9492	0.9382	1.0112
Fe K	0.10	0.03	0.03	9.30	16.11	0.9646	0.9512	1.0515
