# Supplementary material for: Boring life: early colony formation and growth in the endolithic bryozoan genus Penetrantia Silén, 1946
Source: Zoological Lett. 2024 Jun 14;10:10. doi: 10.1186/s40851-024-00234-z (PMC11179354; doi:10.1186/s40851-024-00234-z)
Supplement: Supplementary file 3 — Supplementary Material 3 [file 40851_2024_234_MOESM3_ESM.pdf]

EDAX APEX *Penetrantia japonica*

2023-09-21

Author: CIUS EM - Lab

Creation: 09/21/2023 7:56:16 AM

Sample Name: 1

1

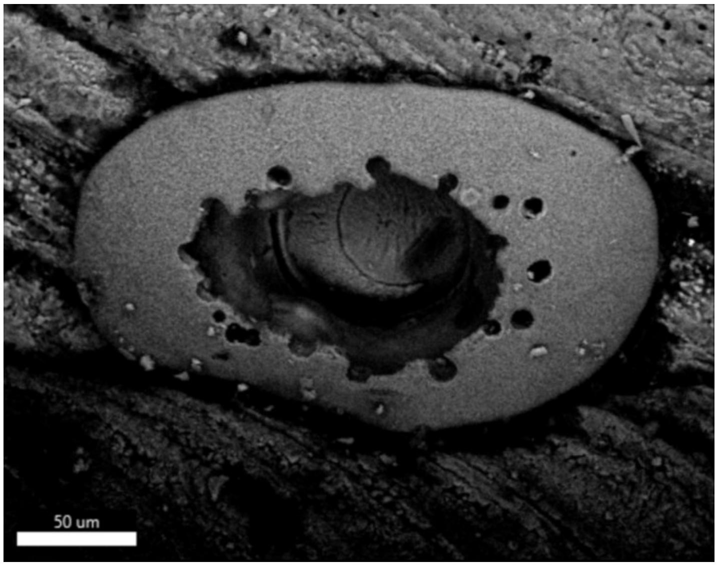

Image

Live Map 1

ElementOverlay

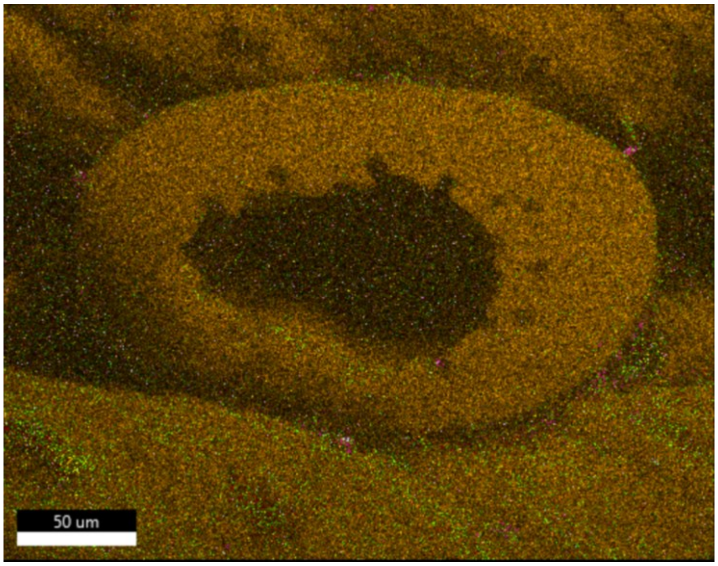

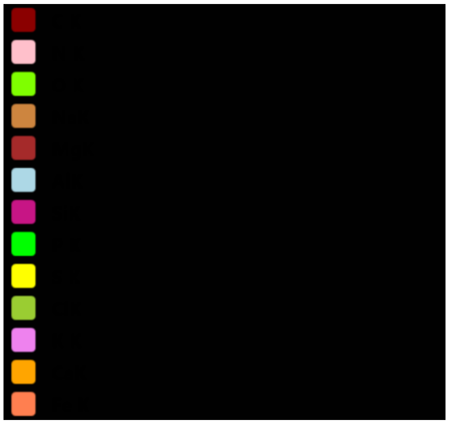

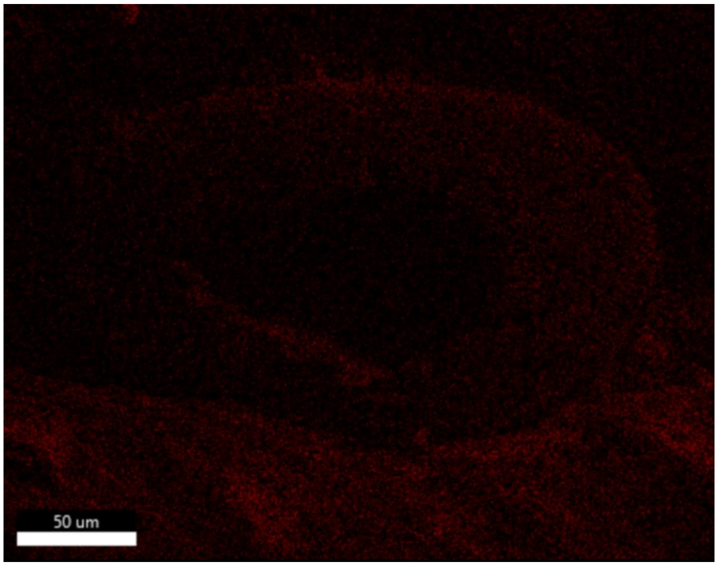

C K_ROI (10)

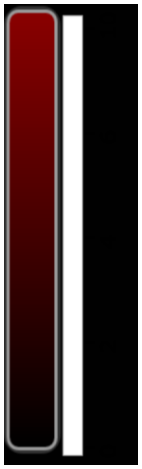

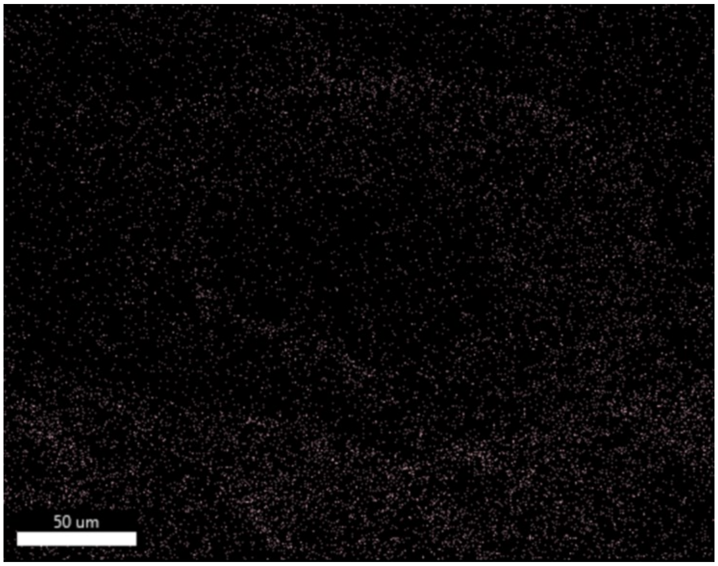

N K_ROI (4)

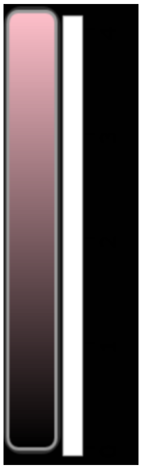

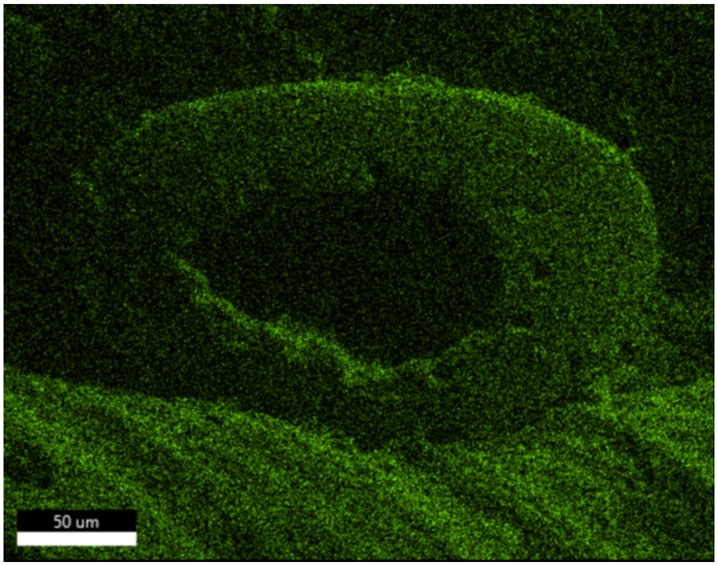

O K_ROI (12)

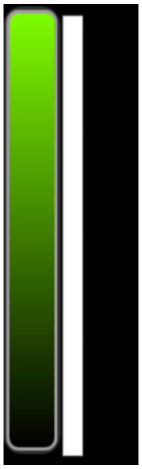

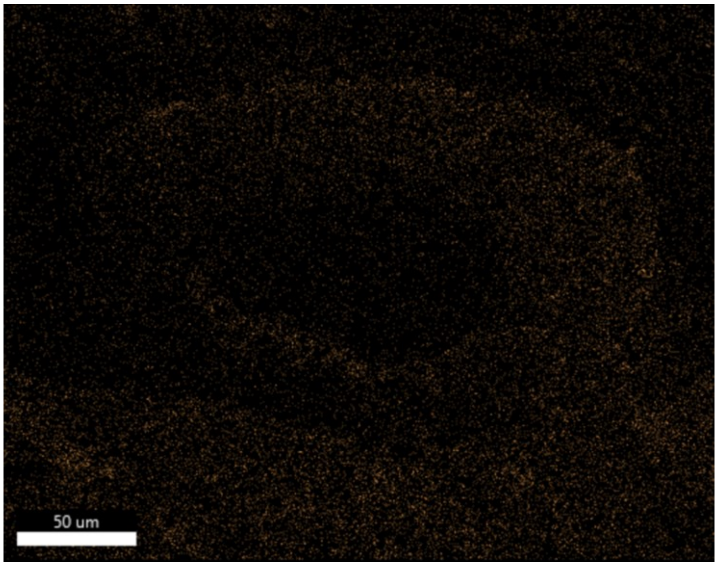

NaK_ROI (6)

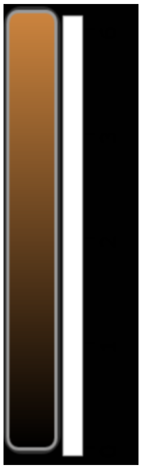

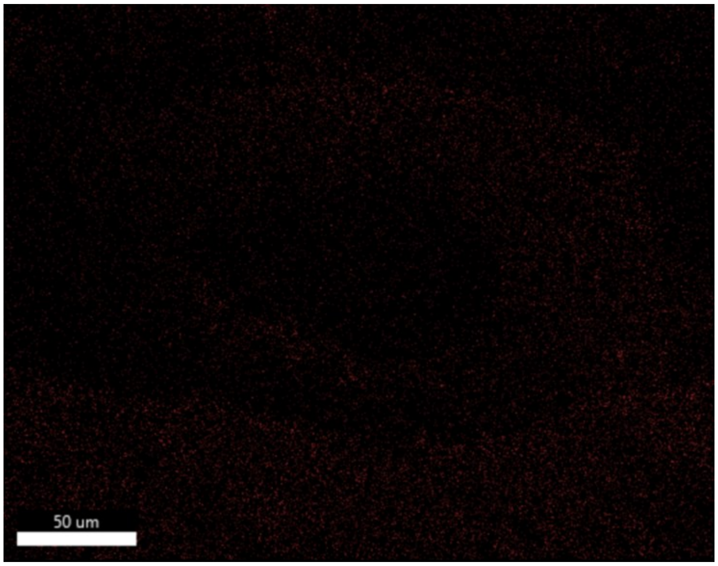

MgK_ROI (6)

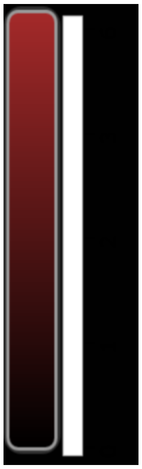

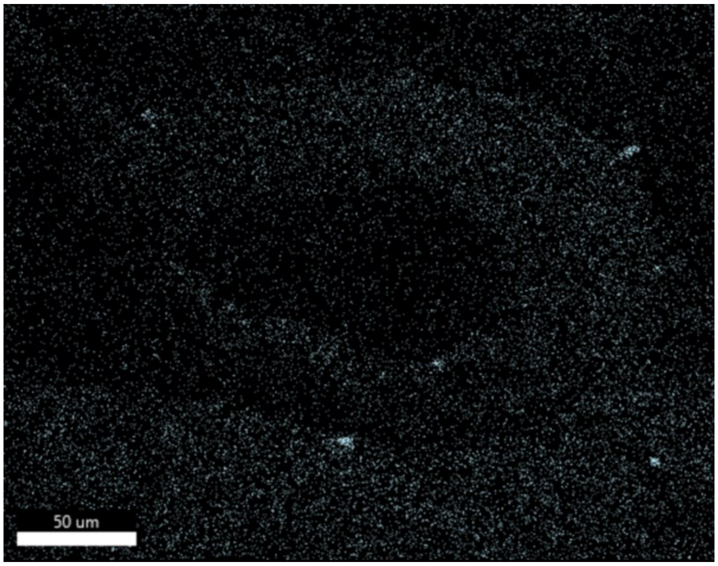

AlK_ROI (6)

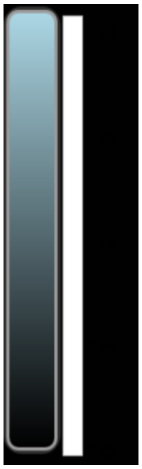

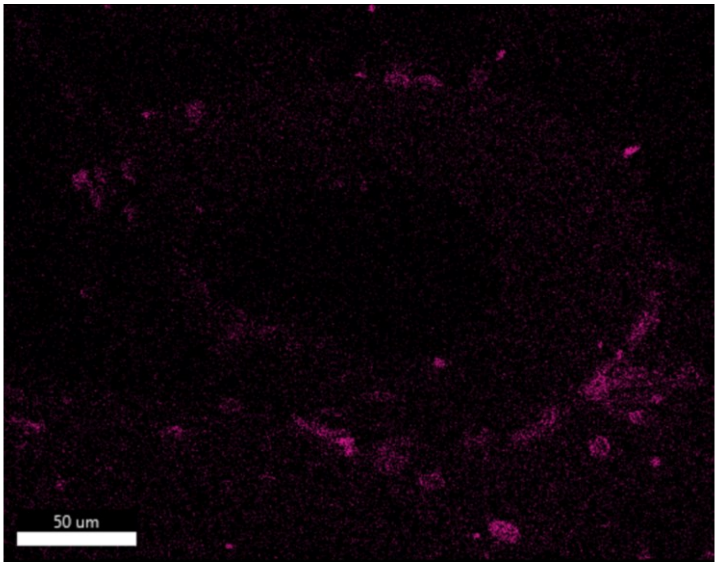

SiK_ROI (14)

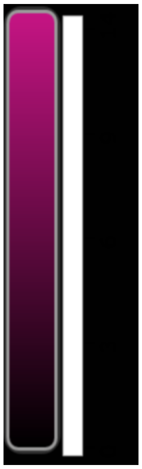

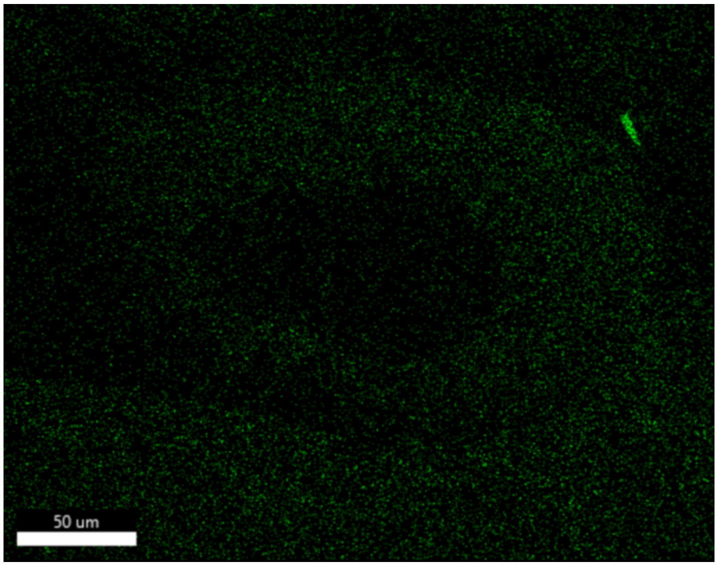

P K_ROI (6)

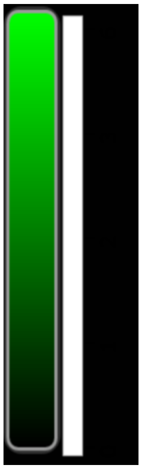

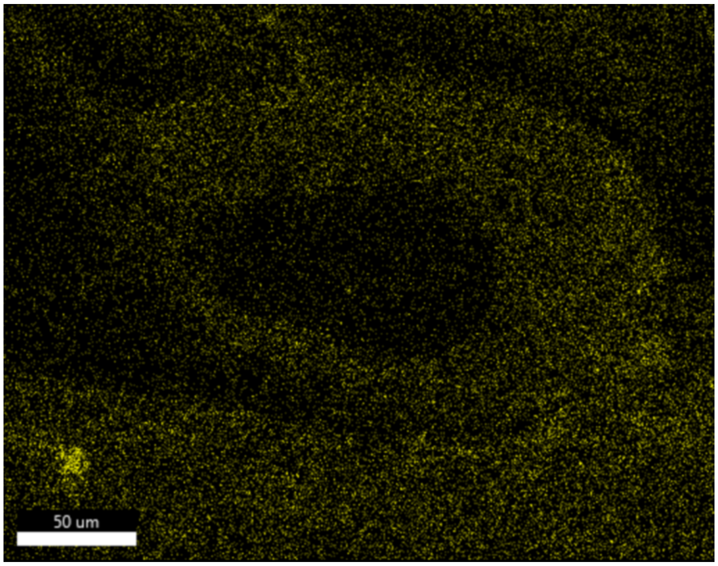

S K_ROI (7)

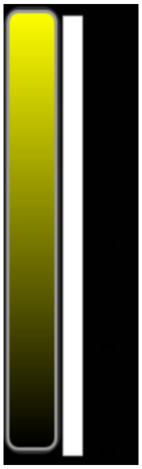

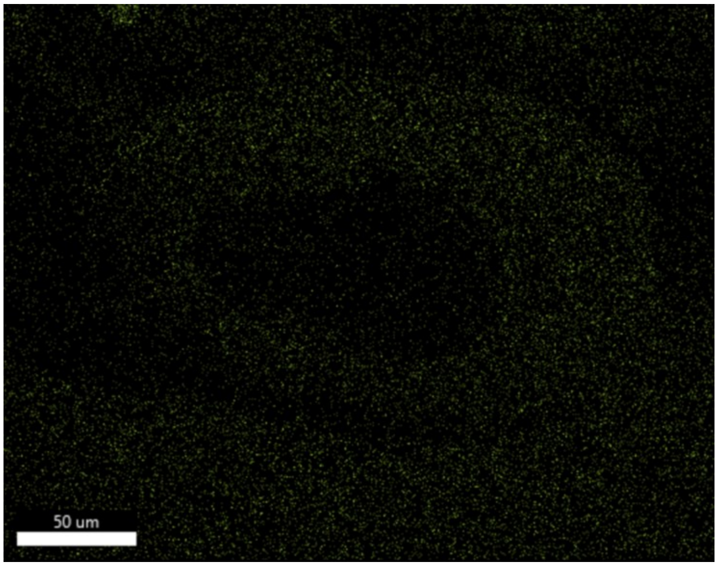

CIK_ROI (5)

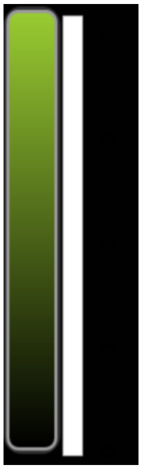

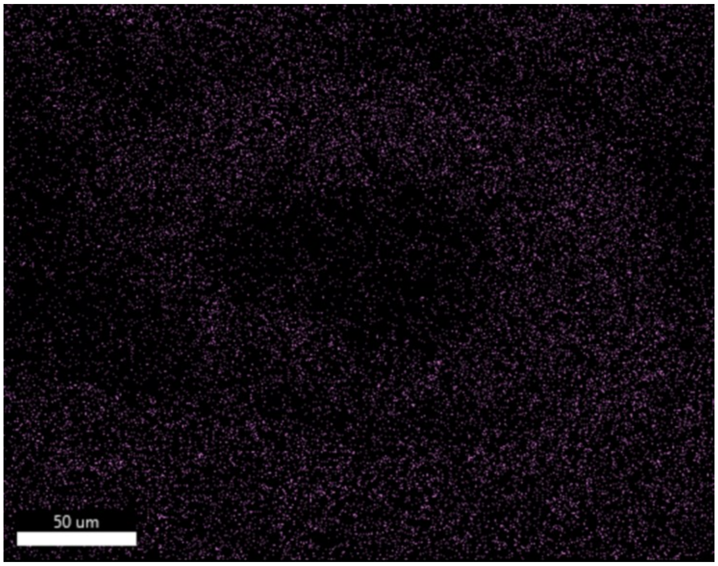

K K_ROI (7)

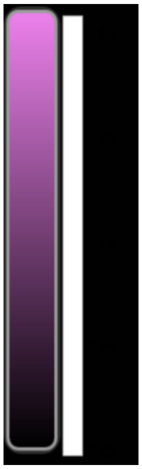

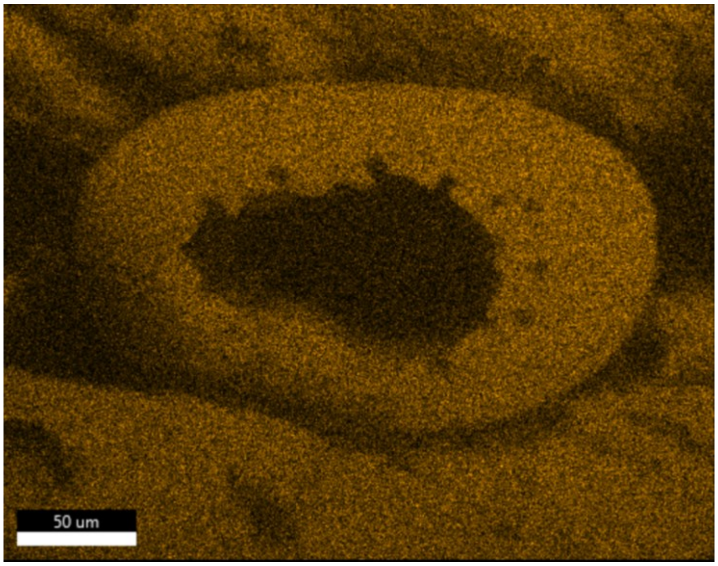

CaK_ROI (32)

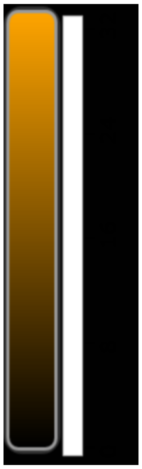

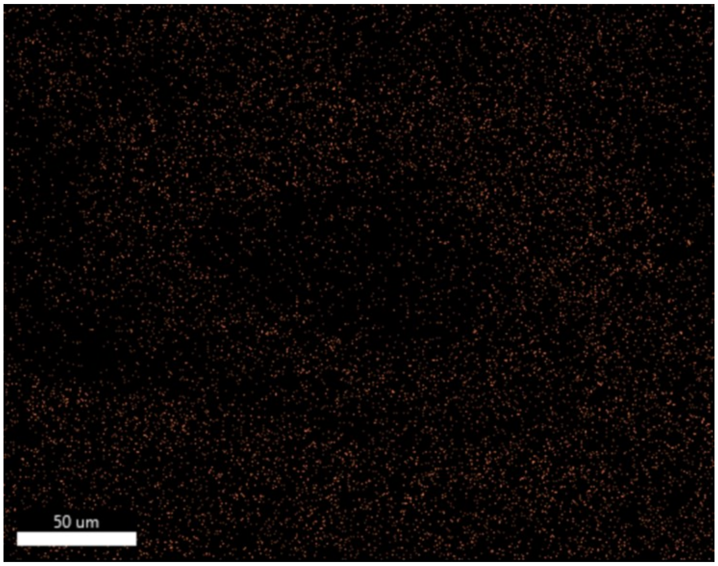

Fe K_ROI (5)

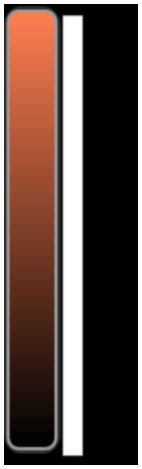

kV: 20	Mag:430	Takeoff: 35.1	Live Time(s): 655.4	Amp Time(µs): 7.68	Resolution:(eV)127.4

Sum Spectrum

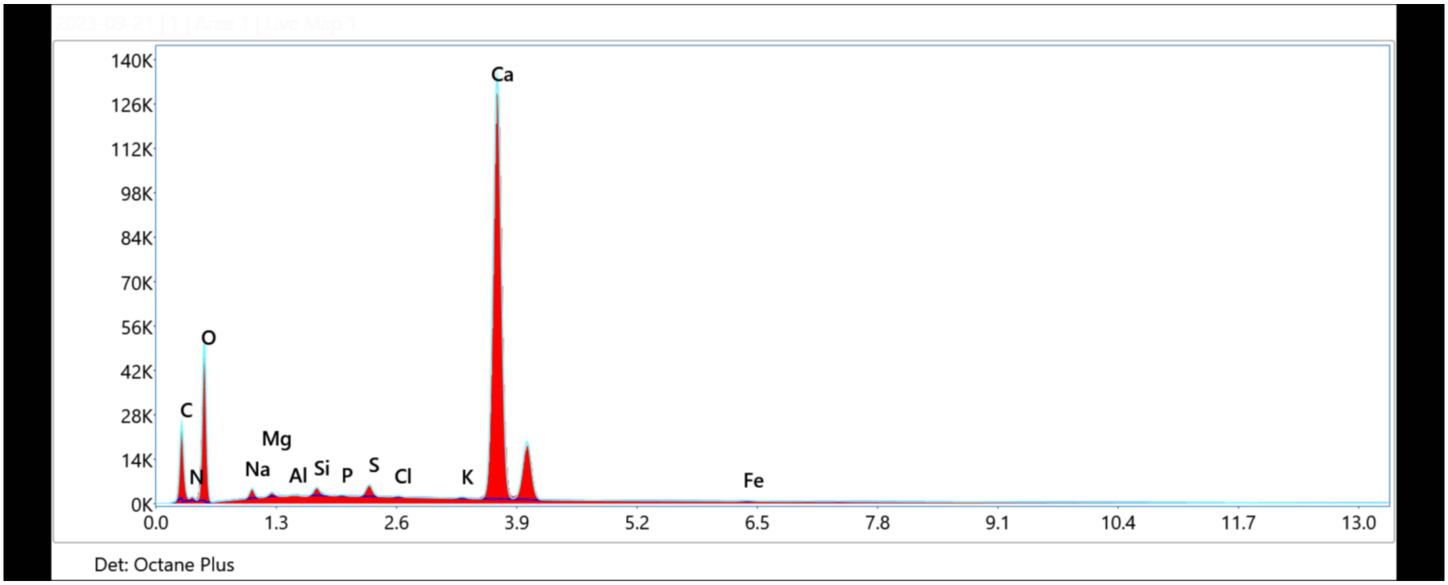

Quant Results

Element	Weight %	Auto MDL	Atomic %	Net Int.	Error %	R	A	F
C K	21.22	0.14	31.01	153.69	10.07	0.9048	0.1234	1.0000
N K	3.45	0.30	4.33	8.24	13.85	0.9107	0.0385	1.0000
O K	47.52	0.09	52.13	420.27	10.39	0.9155	0.0706	1.0000
Na K	0.70	0.03	0.53	30.90	10.23	0.9276	0.2206	1.0031
Mg K	0.15	0.02	0.11	13.10	10.53	0.9313	0.3514	1.0056
Si K	0.24	0.01	0.15	31.16	6.65	0.9380	0.6206	1.0164
P K	0.03	0.02	0.02	4.11	21.78	0.9411	0.7234	1.0273
S K	0.36	0.01	0.20	49.20	5.11	0.9441	0.8019	1.0428
Cl K	0.04	0.01	0.02	5.37	14.85	0.9469	0.8551	1.0681
K K	0.06	0.02	0.03	7.28	17.94	0.9522	0.9242	1.1608
Ca K	26.12	0.02	11.44	2234.38	1.80	0.9548	0.9444	1.0124
Fe K	0.10	0.04	0.03	4.40	25.26	0.9688	0.9602	1.0568
