# Supplementary material for: Boring life: early colony formation and growth in the endolithic bryozoan genus Penetrantia Silén, 1946
Source: Zoological Lett. 2024 Jun 14;10:10. doi: 10.1186/s40851-024-00234-z (PMC11179354; doi:10.1186/s40851-024-00234-z)
Supplement: Supplementary file 7 — Supplementary Material 7 [file 40851_2024_234_MOESM7_ESM.docx]

**Supplementary material**

**Additional file 1: Fig. S1.** Experimental setup Guam and Roscoff.

**Additional file 2: File S2.** Energy dispersive X-ray analysis (EDX) for element analysis of apertural rims and opercula in *Penetrantia clionoides* from Guam. Indicating the assimilation of calcium carbonate (CaCO₃) within both structures.

**Additional file 3:** **File S3.** Energy dispersive X-ray analysis (EDX) for element analysis of apertural rims and opercula in *Penetrantia japonica* from Japan. Indicating the assimilation of calcium carbonate (CaCO₃) within both structures.

**Additional file 4: Movie 1**. Video of lophophore protrusion of ancestrula in *Penetrantia clionoides* from Guam.

**Additional file 5: Movie 2**. Time-lapse video of colony growth in *Penetrantia clionoides* from Guam.

**Additional file 6: Movie 3.** Time-lapse video of colony growth in *Penetrantia* sp. from Roscoff, France.
